# Profiling the cancer-prone microenvironment in a zebrafish model for MPNST

**DOI:** 10.1038/s41388-024-03210-1

**Published:** 2024-11-07

**Authors:** Cheryl Cero, John S. House, Vincenzo Verdi, Jordan L. Ferguson, Dereje D. Jima, Aubrie A. Selmek, Olivia M. Patania, Jennifer E. Dwyer, Bih-Rong Wei, Dillon T. Lloyd, Heather R. Shive

**Affiliations:** 1 Laboratory of Cancer Biology and Genetics, National Cancer Institute, National Institutes of Health, Bethesda, MD USA; 2 Division of Cancer Biology, Cancer Cell Biology Branch, National Cancer Institute, National Institutes of Health, Bethesda, MD USA; 3 Biostatistics and Computational Biology Branch, National Institute of Environmental Health Sciences, National Institutes of Health, Durham, NC USA; 4 State Laboratory of Public Health, North Carolina Department of Health and Human Services, Raleigh, NC USA; 5 Center of Human Health and the Environment and Bioinformatics Research Center, North Carolina State University, Raleigh, NC USA; 6 Department of Veterinary Biosciences, College of Veterinary Medicine, The Ohio State University, Columbus, OH USA; 7 StageBio, Frederick, MD USA; 8 Bioinformatics Research Center, North Carolina State University, Raleigh, NC USA

**Keywords:** Cancer microenvironment, Cancer genomics, Sarcoma, Cancer models

## Abstract

Microenvironmental contributions to soft tissue sarcoma progression are relatively undefined, particularly during sarcoma onset. Use of animal models to reveal these contributions is impeded by difficulties in discriminating between microenvironmental, precancerous, and cancer cells, and challenges in defining a precancerous microenvironment. We developed a zebrafish model that allows segregation of microenvironmental, precancerous, and cancerous cell populations by fluorescence-activated cell sorting. This model has high predilection for malignant peripheral nerve sheath tumor (MPNST), a type of soft tissue sarcoma that exhibits rapid, aggressive growth. Using RNA-seq, we profiled the transcriptomes of microenvironmental, precancerous, and cancer cells from our zebrafish MPNST model. We show broad activation of inflammation/immune-associated signaling networks, describe gene expression patterns that uniquely characterize the transition from precancerous to cancer ME, and identify macrophages as potential contributors to microenvironmental phenotypes. We identify conserved gene expression changes and candidate genes of interest by comparative genomics analysis of MPNST versus benign lesions in both humans and zebrafish. Finally, we functionally validate a candidate extracellular matrix protein, periostin (POSTN), in human MPNST. This work provides insight into how the microenvironment may regulate MPNST initiation and progression.

## Introduction

The tumor microenvironment directly impacts cancer cell survival, growth, invasion, and metastasis. The precancerous microenvironment is proposed to be equally important during cancer initiation [1, 2]. However, specific interactions between microenvironmental cells and incipient cancer cells during cancer onset are challenging to characterize. Furthermore, genetic and molecular events that occur during the transition from precancerous to cancer microenvironment are not defined.

Animal models for heritable cancer syndromes are uniquely suited for identifying microenvironmental factors that support carcinogenesis. In these models, cancers are caused by known genetic mutations and occur in a predictable temporal and tissue-specific manner. However, use of animal models to profile the microenvironment for cancer-associated gene expression patterns necessitates a method for distinguishing microenvironmental cells from precancerous or cancer cells.

To overcome this requisite, we developed a zebrafish model that enabled us to partition microenvironmental, precancerous, and cancer cell populations into mutually exclusive groups by fluorescence-activated cell sorting using a reporter construct to identify cancer cells and potential precancerous cells. This model exhibits high predilection for malignant peripheral nerve sheath tumor (MPNST) [3], a type of soft tissue sarcoma with a particularly poor prognosis due to aggressive growth, limited response to conventional treatment, and ineffective targeted therapies [4–7]. In our model, MPNST is caused by combined heritable mutations in the tumor suppressor genes *TP53* and *BRCA2* [8–10]. MPNSTs in this model preferentially arise in a discrete anatomic location within a predictable timeframe [9, 10], facilitating definition of the precancerous microenvironment.

We profiled transcriptomes of precancerous and cancer microenvironments from our zebrafish MPNST model by gene expression and ontology analyses. We demonstrated broad activation of inflammatory and immune-associated signaling networks in precancerous and cancer microenvironments and identified gene expression patterns that uniquely define cancer versus precancerous microenvironments. Cancers contained numerous presumptive macrophages that were frequently located in the periphery and at invasive margins. Markers for both M1 and M2 macrophage polarization were upregulated in precancerous and cancer microenvironments, suggesting the presence of a mixed macrophage population during sarcomagenesis. Using a comparative genomics approach, we identified conserved gene expression differences in MPNST versus benign samples in human and zebrafish and confirmed expression of select extracellular matrix proteins in human and zebrafish MPNST. Finally, we functionally validated the matricellular protein periostin (POSTN) as a contributor to human MPNST cell growth. This work identifies distinguishing characteristics of the cancer-prone cellular microenvironment that potentially influence MPNST initiation and progression in vertebrates.

## Results

### Isolation and analysis of the cellular microenvironment from cancer-prone tissues using a zebrafish model

**Fig. 1:**
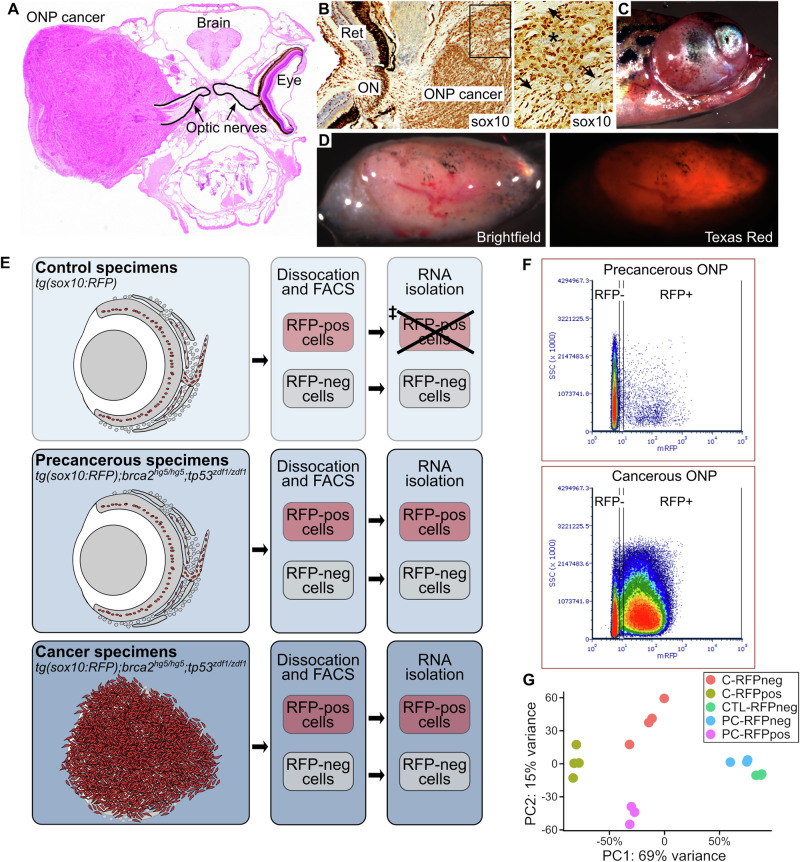
Use of a *tg(sox10:RFP);brca2*^*hg5/hg5*^*;tp53*^*zdf1/zdf1*^ zebrafish model to isolate and analyze the cellular component of a cancer-prone microenvironment. **A** The optic nerve pathway (ONP) is a cancer-prone site in *brca2*^*hg5/hg5*^*;tp53*^*zdf1/zdf1*^ zebrafish. **B** Zebrafish ONP cancers exhibit ubiquitous sox10 expression (brown chromogen). Asterisk, blood vessel containing sox10-negative erythrocytes; arrows, fragments of optic nerve. Ret, retina; ON, optic nerve. **C**, **D** Zebrafish carrying a sox10-RFP reporter construct (*tg(sox10:RFP);brca2*^*hg5/hg5*^*;tp53*^*zdf1/zdf1*^) develop RFP-expressing cancers. **E** Experimental design showing experimental cohorts and workflow for tissue collection and RNA isolation. RFP-positive cells are shown in red. Remaining areas shaded in gray are composed of RFP-negative cells. ^‡^RNA was of insufficient quantity for RNAseq analysis. **F** RFP-positive and RFP-negative cell fractions were collected from isolated ONP tissues by fluorescence-activated cell sorting (FACS). FACS analysis of control (not shown) and precancerous ONP samples showed similar distributions of RFP-positive and RFP-negative cell populations. The full gating strategy, including the panels shown in (**F**), is in Fig. S1. **G** Principal component analysis of samples analyzed by RNA-seq.

We previously showed that zebrafish with mutations in *brca2* and *tp53* develop soft tissue sarcomas with histologic and immunohistochemical features of MPNST [3, 8, 10]. Although mutations in *TP53* and *BRCA2* are uncommon in human MPNST [11–13], ERK and AKT activation occur frequently [14–16] and are detectable in cancers from our zebrafish model (Fig. S1A). The optic nerve pathway (ONP) is a cancer-prone site in our model, with particularly high cancer incidence in this location in *brca2*^*hg5/hg5*^*;tp53*^*zdf1/zdf1*^ zebrafish [9, 10]. Tissues within the ONP are circumscribed by the infraorbital bones that surround the eye and associated soft tissues [17, 18] (Fig. 1A), allowing discrete and consistent collection of tissues from this location (Fig. S2A–C). ONP cancers in *brca2*^*hg5/hg5*^*;tp53*^*zdf1/zdf1*^ zebrafish are further defined by widespread sox10 expression (Figs. 1B, S3, and Kouprianov et al. [8]) and the precancerous ONP in *brca2*^*hg5/hg5*^*;tp53*^*zdf1/zdf1*^ frequently exhibits aberrant proliferation of sox10-positive cells [8]. Therefore, we introduced a sox10:RFP reporter construct [19] to generate *tg(sox10:RFP);brca2*^*hg5/hg5*^*;tp53*^*zdf1/zdf1*^ zebrafish (Fig. 1C). Sox10-expressing (RFP-positive) cells in the ONP from zebrafish of this genotype constitute a pool of cells from which a malignant clone is likely to emerge [8] and are subsequently referred to as “potential precancerous cells”. Cancers from this zebrafish cohort highly express RFP (Fig. 1D).

We used RFP expression to segregate and analyze cancer and potential precancerous cells from the cellular component of the ONP microenvironment (Fig. 1E). The experimental cohorts in Table 1 were used to collect ONP samples from control (*tg(sox10:RFP*)), precancerous (*tg(sox10:RFP);brca2*^*hg5/hg5*^*;tp53*^*zdf1/zdf1*^*)*, and cancer (*tg(sox10:RFP);brca2*^*hg5/hg5*^*;tp53*^*zdf1/zdf1*^) groups. We previously showed a mean age at tumor onset of 8.7 months in *brca2*^*hg5/hg5*^*;tp53*^*zdf1/zdf1*^ zebrafish [10], while aberrantly proliferative cells arise in the precancerous ONP as early as 4 months of age [8]. Control and precancerous ONP samples were pooled from five individual zebrafish per replicate, while ONP cancer samples were collected from individual cancer-bearing zebrafish (Table 1). We analyzed dissociated cells from ONP samples by fluorescence-assisted cell sorting (Figs. 1F and S1B, C) and collected RFP-positive and RFP-negative cell populations for RNA-seq analysis. In both precancerous (Fig. 1F) and control (not shown) ONP samples, most cells were RFP-negative. In comparison, most cells from ONP cancer specimens were RFP-positive (Fig. 1F). RFP-negative fractions were analyzed for all three experimental cohorts. We were unable to isolate RNA of sufficient quantity and quality from the RFP-positive fraction of control samples, and therefore only RFP-positive fractions from precancerous and cancer samples were analyzed. RNA-seq analysis generated ~35-45 million uniquely mapped reads per sample (Fig. S4A) and principal component analysis of replicates revealed distinct clustering within experimental cohorts (Fig. 1G, Fig. S4B). To facilitate subsequent bioinformatics analyses, zebrafish gene names were converted to known human orthologues using a publicly available dataset to generate a “humanized” gene list (see Methods).

**Table 1:** Study populations used for RNAseq analysis of the cellular microenvironment in the zebrafish optic nerve pathway (ONP).

Cohort	# Males	# Females	Age (mo)
Precancerous ONP (*tg(sox10:RFP);brca2*^*hg5/hg5*^*;tp53*^*zdf1/zdf1*^)^a^			
Replicate 1 (*n* = 5)	4	1	4.5
Replicate 2 (*n* = 5)	2	3	4.5
Replicate 3 (*n* = 5)	3	2	4.7
Control ONP (*tg(sox10:RFP)*)^a^			
Replicate 1 (*n* = 5)	3	2	4.6
Replicate 2 (*n* = 5)	3	2	5.0
Replicate 3 (*n* = 5)	2	3	5.0
ONP cancers (*tg(sox10:RFP);brca2*^*hg5/hg5*^*;tp53*^*zdf1/zdf1*^)^b^			
Ocular cancer 1 (OD)	--	1	7.5
Ocular cancer 2 (OD)	1	--	8.1
Ocular cancer 3 (OS)	1	--	8.1
Ocular cancer 4 (OD)	--	1	10.4

*Mo* months, *OD* right side, *OS* left side.

^a^ONP tissues from both the right and left sides were collected and pooled from five zebrafish for each replicate.

^b^ONP cancers were collected and analyzed individually.

### Pathway and gene set enrichment analyses of precancerous and cancer microenvironments suggests activation of immune/inflammatory networks

**Fig. 2:**
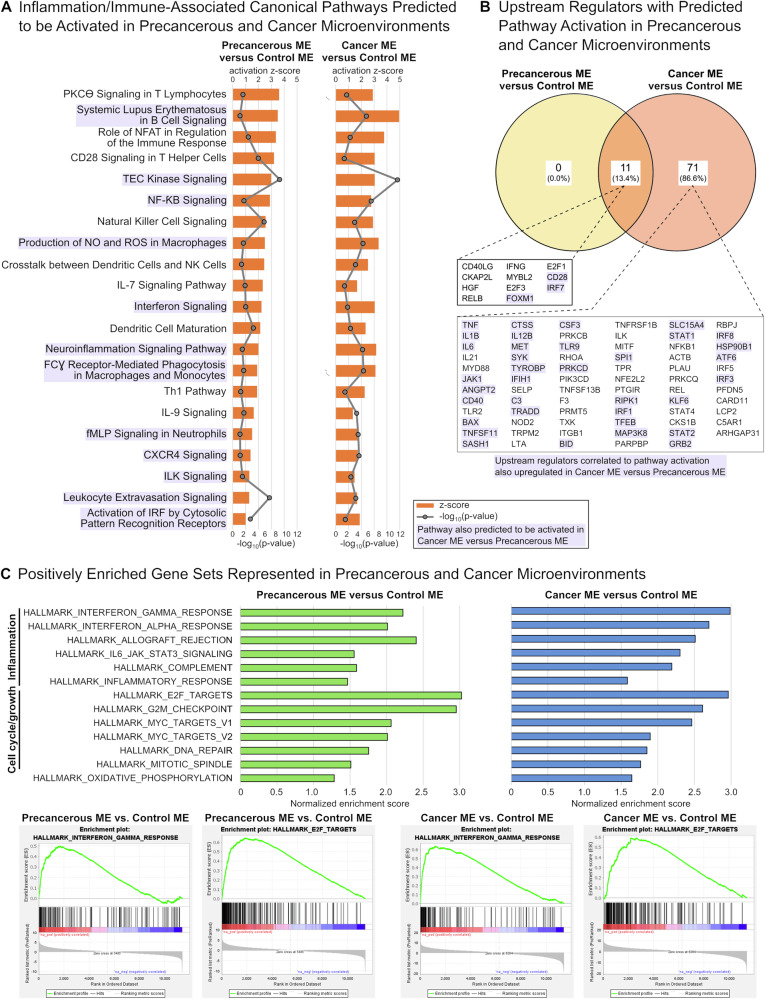
Pathway and gene set enrichment analyses suggest activation of pro-inflammatory and pro-growth signaling in precancerous and cancer cellular microenvironments. **A** Inflammation and immune-associated canonical pathways identified by Ingenuity Pathways Analysis (IPA) that were predicted to be activated for comparisons of precancerous versus control and cancer versus control microenvironments (MEs). Orange bars represent activation z-score with predicted pathway activation; grey lines represent −log_10_(enrichment *p* value); purple shading indicates pathways that were also predicted to be activated in the comparison of cancer versus precancerous microenvironments (Table S1). **B** Genetic upstream regulators associated with predicted pathway activation as identified by IPA that were significantly upregulated in each comparison. Purple shading indicates genetic upstream regulators associated with predicted pathway activation pathways that were also significantly upregulated in the comparison of cancer versus precancerous MEs (Table S2). **C** Gene set enrichment analysis (GSEA) identified 13 hallmark gene sets with positive normalized enrichment scores (NES) in both precancerous and cancer MEs versus the control ME. All 13 gene sets also had positive NES in the comparison of cancer versus precancerous MEs (Table S3). These commonly enriched hallmark gene sets were predominantly associated with inflammation or cell cycle/cell growth processes.

Datasets were analyzed using Ingenuity Pathways Analysis (IPA) and significantly affected pathways for each comparison were identified (Table S1) and segregated based on the predicted direction of activity (Fig. S5). In precancerous and cancer microenvironments, enriched canonical pathways with predicted pathway activation included a broad and diverse array of pathways associated with inflammation, immune cell signaling, and chronic inflammatory conditions (Figs. 2A and S5B).

We next used IPA to evaluate upstream transcriptional regulators that correlated to observed changes in gene expression for each comparison (Table S2). We refined this analysis to identify genes classified as upstream regulators that were associated with predicted pathway activation in our data set and were also significantly upregulated in the precancerous and/or cancer microenvironment versus the control microenvironment (Fig. 2B). In agreement with the above findings, many upstream regulators correlated to pathway activation in precancerous or cancer microenvironments were pro-inflammatory signaling molecules. This included zebrafish orthologues for interferon gamma (*IFN-γ*), interleukin 1 beta (*IL1B*), interleukin 6 (*IL6*), and tumor necrosis factor alpha (*TNF-α*) (Fig. 2B). Also enriched were positive cell cycle regulators such as zebrafish orthologues for the E2F transcription factors *E2F1* and *E2F3* (Fig. 2B and Table S2).

We used Gene Set Enrichment Analysis (GSEA) to identify hallmark gene sets enriched in precancerous and cancer microenvironments (Table S3). 13 hallmark gene sets had a positive normalized enrichment score (NES) in the comparisons of both precancerous and cancer microenvironments versus the control microenvironment (Fig. 2C). These gene sets also had a positive NES in the comparison of cancer versus precancerous microenvironments. Nearly all were associated with inflammation (6 of 13 gene sets) or cell cycle progression and cell growth (6 of 13 gene sets) **(**Fig. 2C).

### Pathway and gene set enrichment analyses identify expression profile changes that uniquely define the cancer microenvironment

**Fig. 3:**
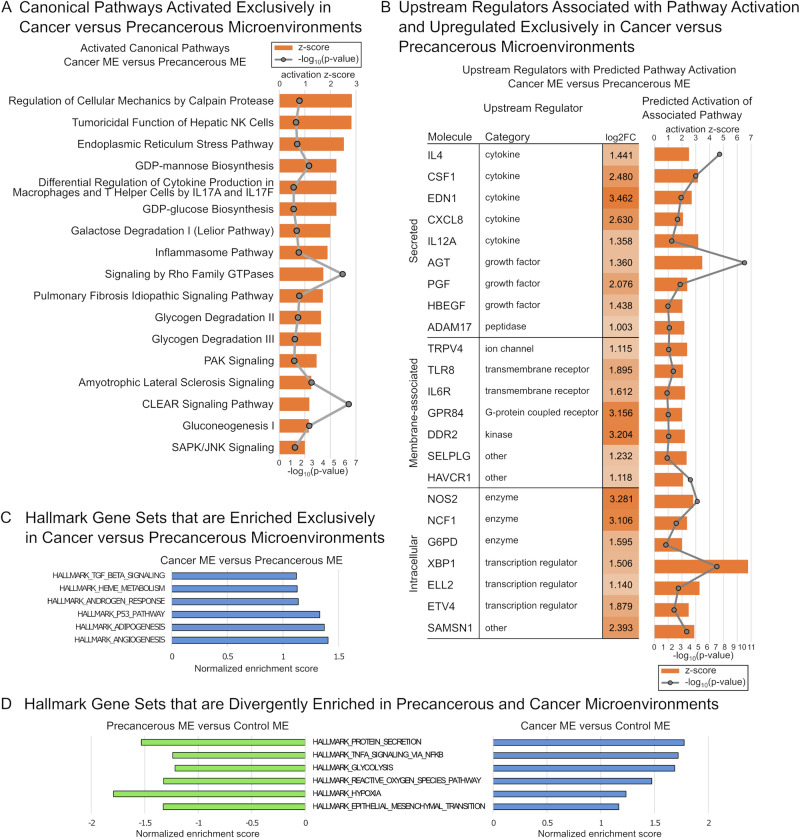
Pathway and gene set enrichment analyses identify gene expression changes that uniquely characterize the cancer versus precancerous microenvironment. **A** Canonical pathways identified by Ingenuity Pathways Analysis (IPA) that were predicted to be activated exclusively in the comparison of cancer versus precancerous microenvironments (MEs). **B** Genetic upstream regulators associated with predicted pathway activation as identified by IPA that were significantly upregulated exclusively in the comparison of cancer versus precancerous MEs. **C** Gene set enrichment analysis (GSEA) identifies six hallmark gene sets with a positive normalized enrichment score (NES) exclusively in the comparison of cancer versus precancerous MEs. **D** GSEA identifies 6 hallmark gene sets with a negative NES in the comparison of precancerous versus control MEs, but a positive NES in the comparison of cancer versus control MEs.

To assess changes that may characterize the progression from precancerous to cancer microenvironment, we used IPA and GSEA to identify gene expression patterns that are unique to the cancer microenvironment. IPA identified 17 enriched canonical pathways with predicted pathway activation that were exclusive to the comparison of cancer versus precancerous microenvironments (Fig. 3A and Table S1). A number of enriched pathways are associated with cellular metabolism and include both synthetic and degradative processes (e.g., gluoconeogenesis, glycogen degradation, and lysosomal function).

We next assessed upstream transcriptional regulators that correlated to our observed changes in gene expression specifically in the comparison of cancer versus precancerous microenvironments (Table S2). This analysis focused on genes classified as upstream regulators that were associated with predicted pathway activation exclusively in the comparison of cancer versus precancerous microenvironments and were also significantly upregulated in the comparison of cancer versus precancerous microenvironments (Fig. 3B). The 23 upstream regulators identified by this analysis were predominated by secreted factors and membrane-associated signaling molecules. They included molecules that directly or indirectly regulate inflammatory cell growth and behavior, including zebrafish orthologues for colony stimulating factor 1 (*CSF1*), CXC motif chemokine ligand 8 (*CXCL8*), P-selectin glycoprotein ligand-1 (PSGL-1, encoded by *SELPLG*), and ADAM metallopeptidase domain 17 (*ADAM17*) (Fig. 3B).

GSEA identified 6 hallmark gene sets with a positive NES exclusively in the comparison of cancer versus precancerous microenvironment (Fig. 3C and Table S3). We also identified 6 hallmark gene sets that were divergently enriched in precancerous and cancer microenvironments, i.e., gene sets were associated with a negative NES in the comparison of precancerous versus control microenvironments and a positive NES in the comparison of cancer versus control microenvironments (Fig. 3D and Table S3). Positively enriched hallmark gene sets that distinguished the cancer microenvironment include signaling networks such as TNF-alpha signaling; metabolism-associated processes such as glycolysis; and microenvironmental conditions that include hypoxia, adipogenesis, and angiogenesis (Fig. 3C, D).

### Macrophages may contribute to precancerous and cancer microenvironmental phenotypes

**Fig. 4:**
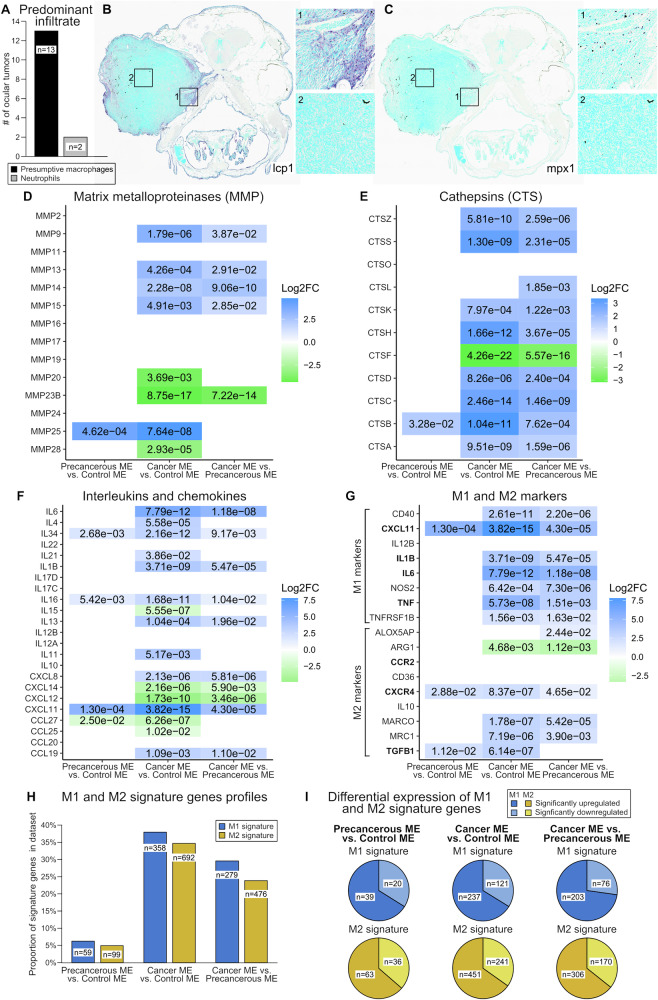
Presumptive macrophages may contribute to precancerous and cancer microenvironmental phenotypes. Human orthologues for zebrafish genes are shown. Gene expression data reflects comparisons of precancerous versus control, cancer versus control, and cancer versus precancerous cellular microenvironments (ME). Numerical values in panels **D**–**G** show adjusted *p*-values for the comparisons and color shading indicates log_2_ fold change (FC) values. **A** Identification of predominant inflammatory cell type in zebrafish ONP cancers as defined by lcp1 expression (presumptive macrophages) and mpx1 expression (neutrophils). **B** Lcp1-expressing presumptive macrophages (purple chromogen) are abundant and predominantly localize to peripheral margins and invasive edges. **C** Mpx1-expressing neutrophils (purple chromogen) are present in low numbers. **D** Matrix metalloproteinase (MMP) gene expression profile. **E** Cathepsin (CTS) gene expression profile. **F** Interleukin and chemokine gene expression profile. **G** Expression of known markers for mammalian M1 and M2 phenotypes. Gene names in bold have been identified previously as M1 and M2 markers in zebrafish [34, 36]. **H** Proportions of differentially expressed M1 and M2 signature genes [37]. **I** Proportions of genes identified in panel (**H**) that were significantly up- or downregulated.

Given the pro-inflammatory gene expression profiles for the precancerous and cancer microenvironments, we analyzed zebrafish ONP cancers for the presence of macrophages and neutrophils. 15 ONP cancers from *brca2*^*hg5/hg5*^*;tp53*^*zdf1/zdf1*^ that were described in a previous study [9] were analyzed for expression of myeloperoxidase (mpx1) and l-plastin (lcp1) (Figs. 4A–C and S1D–G). While mpx1 is considered neutrophil-specific in zebrafish, some studies suggest that both neutrophils and macrophages can express lcp1 and are differentiated by presence or absence of mpx1 expression [20–23]. However, lcp1 has been used as a specific marker for zebrafish macrophages [23–25]. These differences may reflect different methodologies used for cell identification and/or differences in developmental stage. In the current study, we observed limited overlap of lcp1- and mpx1-expressing cells in serial sections of ONP cancers, suggesting that most lcp1-positive cells in these specimens are macrophages (Figs. 4B, C and S1F, G). We attempted to confirm this with a reportedly macrophage-specific antibody (mfap4 [26]), but in our hands this antibody did not generate consistent labeling in control tissues. We therefore considered mpx-positive cells to be neutrophils and lcp1-positive cells to be presumptive macrophages.

13 of 15 ONP cancers (87%) were dominated by infiltrating presumptive macrophages, as indicated by the predominance of lcp1-positive cells versus mpx1-positive cells (Fig. 4A–C). In these 13 cancers, lcp1-positive cells were frequently located along the periphery and invasive margins (Fig. 4B). Two of 15 ONP cancers (13%) exhibited relatively greater expression of mpx1 versus lcp1, indicating a predominance of neutrophils (Figs. 4A and S1F, G). In these two cancers, mpx1-positive cells were present primarily within the tumor, while lcp1-positive cells were more abundant at the tumor periphery, and there was limited overlap of cells expressing these markers in serial sections (Fig. S1F, G).

Given the frequent localization of lcp1-positive presumptive macrophages to the invasive edge of zebrafish ONP cancers, we assessed the relative expression of matrix metalloproteinases (MMPs) and cathepsins (CTSs) in precancerous and cancer microenvironments versus the control microenvironment (Fig. 4D, E). The precancerous microenvironment showed significant upregulation of zebrafish orthologues for *MMP25* and *CTSB*, while orthologues for multiple MMPs and CTSs were upregulated in the cancer microenvironment. Notably, downregulation of zebrafish orthologues for *MMP23B*, *MMP28*, and *CTSF* are consistent with downregulated expression of these genes in several human cancer types [27–29]. We also assessed the relative expression of interleukins and chemokines in precancerous and cancer microenvironments versus the control microenvironment (Fig. 4F). Several of these factors were significantly upregulated in the precancerous microenvironment, while numerous pro-inflammatory and pro-tumorigenic interleukins and chemokines [30–33] were upregulated in the cancer microenvironment.

M1/M2 macrophage polarization has been described in zebrafish and carp, and comparative genetic analyses suggest conservation of M1 and M2 gene expression profiles in fish and humans [34–36]. To determine whether precancerous and cancer microenvironments exhibited gene expression profiles consistent with macrophage polarization, we assessed expression of select mammalian markers for M1 polarization (*IL1B*, *IL6*, *TNF*, *CXCL11*, *NOS2*, *TNFRSF1B*, *IL12B*, and *CD40*) and M2 polarization (*IL10*, *MARCO*, *ARG1*, *ALOX5AP*, *MRC1/CD206*, *TGFB1*, and *CD36*) (Fig. 4G). These markers include genes previously used to define M1 and M2 polarization in embryonic zebrafish (M1, orthologues for *IL1B*, *IL6*, *TNF*, and *CXCL11*; M2, orthologues for *TGFB1*, *CCR2*, and *CXCR4*) [34, 36]. Both M1 and M2 markers were significantly upregulated in precancerous and cancer microenvironments, inconsistent with macrophage polarization.

We profiled the expression of validated gene lists for M1 and M2 phenotypes derived from the hybrid mouse diversity panel, which were predictive of macrophage response in various human diseases such as cancer [37]. 944 (74%) of M1 signature genes and 1998 (76%) of M2 signature genes [37] were represented in the humanized gene list derived from zebrafish specimens. Comparison of differentially expressed M1 and M2 signature gene profiles did not indicate clear polarization toward one phenotype in either precancerous or cancer microenvironments (Fig. 4H). The proportions of M1 and M2 signature genes for each comparison were similar, and signature genes were predominantly upregulated (Fig. 4I).

### Cross-species comparative genomics analysis and candidate gene evaluation in human patient samples

**Fig. 5:**
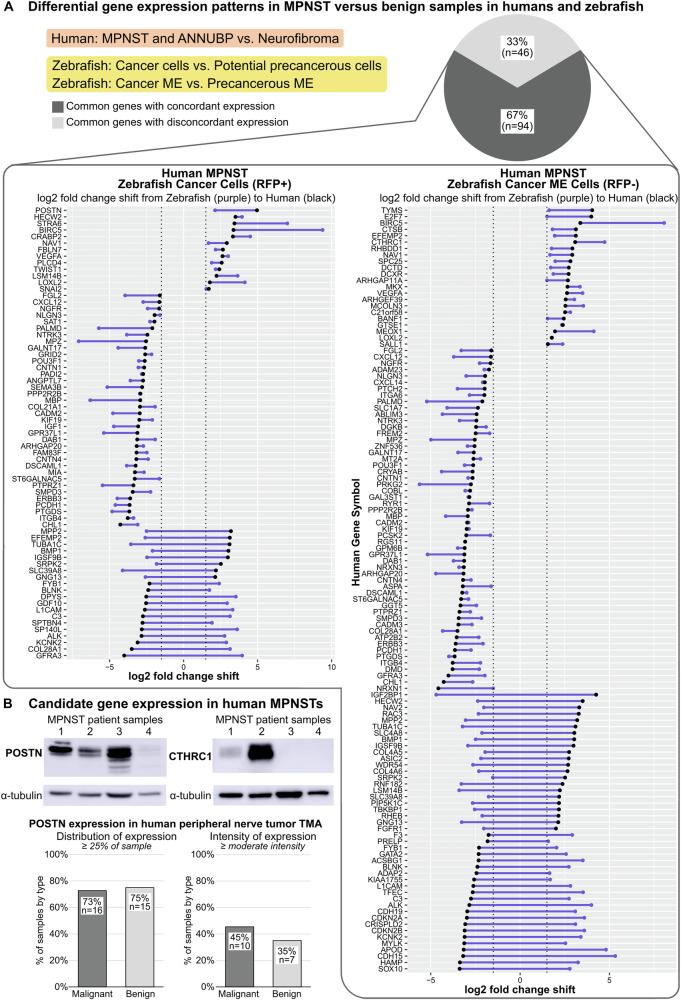
Comparative genomics analysis and candidate gene evaluation in human patient samples identifies potential contributors to MPNST progression. **A** Comparison of gene expression profiles for malignant versus benign samples in humans (MPNST and ANNUBP versus neurofibroma) and zebrafish (cancer cells versus potential precancerous cells; cancer microenvironment (ME) versus precancerous ME). Dots represent log_2_ fold changes per gene for human (black dots) and zebrafish (purple dots) comparisons and connecting lines (purple) depict the relative shift in log_2_ fold change value. **B** Expression of candidate genes POSTN and CTHRC1 in human MPNST specimens detected by Western blotting and immunohistochemistry. ANNUBP, atypical neurofibromatosis neoplasm of unidentified biologic potential; TMA, tissue microarray.

To identify potential conserved contributors to MPNST tumorigenesis, we performed a cross-species comparison of differentially expressed genes in benign versus malignant specimens from human patients and from our zebrafish model. We compared our gene expression data to a previous study [38] reporting gene expression differences in human MPNST and ANNUBP (atypical neurofibromatosis neoplasm of unidentified biologic potential [39]) versus neurofibroma. In performing this analysis, we note that we have not identified neurofibroma or ANNUBP in our zebrafish model, although an aberrant proliferative cell population does arise prior to MPNST onset [8]; the comparison of benign to malignant specimens in humans and zebrafish is thus imperfect. Nonetheless, comparison of gene expression profiles from MPNST versus benign specimens in humans and zebrafish identified 140 genes common to both data sets (Fig. 5A). 67% (*n* = 94) of differentially expressed genes showed concordant expression patterns in human and zebrafish specimens, i.e., up- or downregulated expression in both human and zebrafish MPNST versus their respective benign counterparts. Multiple of these genes have been previously identified as contributors to human MPNST progression, such as *BIRC5* [40], *CRABP2* [41], and *TWIST1* [42].

Periostin (POSTN) and CTHRC1 are extracellular matrix (matricellular) proteins that contribute to progression in multiple human cancer types but have not been studied in MPNST. Both genes are significantly upregulated in MPNSTs in humans and zebrafish versus their respective benign counterparts (Fig. 5A). We assessed POSTN and CTHRC1 expression in human patient-derived MPNST samples and a tissue microarray (TMA) composed of core biopsies from benign and malignant human peripheral nerve tumors (Figs. 5B and S6A–C). POSTN was strongly expressed in 3 of 4 human MPNST samples, while CTHRC1 was strongly expressed in 1 of 4 samples. Semi-quantitative analysis of POSTN expression in the human TMA showed that similar proportions of malignant (73%; *n* = 16 of 22 cores) and benign tumors (75%; *n* = 15 of 20 cores) exhibited POSTN expression in at least 25% of the sample. A higher proportion of malignant tumors (45%; *n* = 10 of 22 cores) showed POSTN expression of at least moderate intensity compared to benign tumors (35%; *n* = 7 of 20 cores).

We also assessed POSTN expression in zebrafish MPNSTs by RNA in situ hybridization (Fig. S7). As POSTN is a duplicated gene in zebrafish, we validated RNA probes for *postna* and *postnb* (Fig. S7A) and analyzed both orthologues in the same zebrafish ONP cancer specimens used for IHC analyses (Figs. 4, S1F, G, and S3). Three of the 15 specimens had insufficient tumor tissue remaining for ISH analysis and thus 12 ONP cancer specimens were analyzed. *postna* expression was detected in 8 of 12 ONP cancers and *postnb* expression was detected in 10 of 12 ONP cancers. There was considerable variability in expression of both orthologues in ONP cancer specimens and moderate levels of expression were observed in only two specimens (Fig. S7B). The remaining *postn*-expressing cancers exhibited low *postnb* expression and very rare foci of positivity for *postna* (Fig. S7C). Although detectable expression was low overall, we noted that foci of *postna* and *postnb* expression were often at the interface of cancer cells and non-cancer tissues located either at the tumor margin or entrapped within the tumor (Fig. S7B, C).

### Functional assessment validates the matricellular protein periostin (POSTN) as a contributor to MPNST growth

**Fig. 6:**
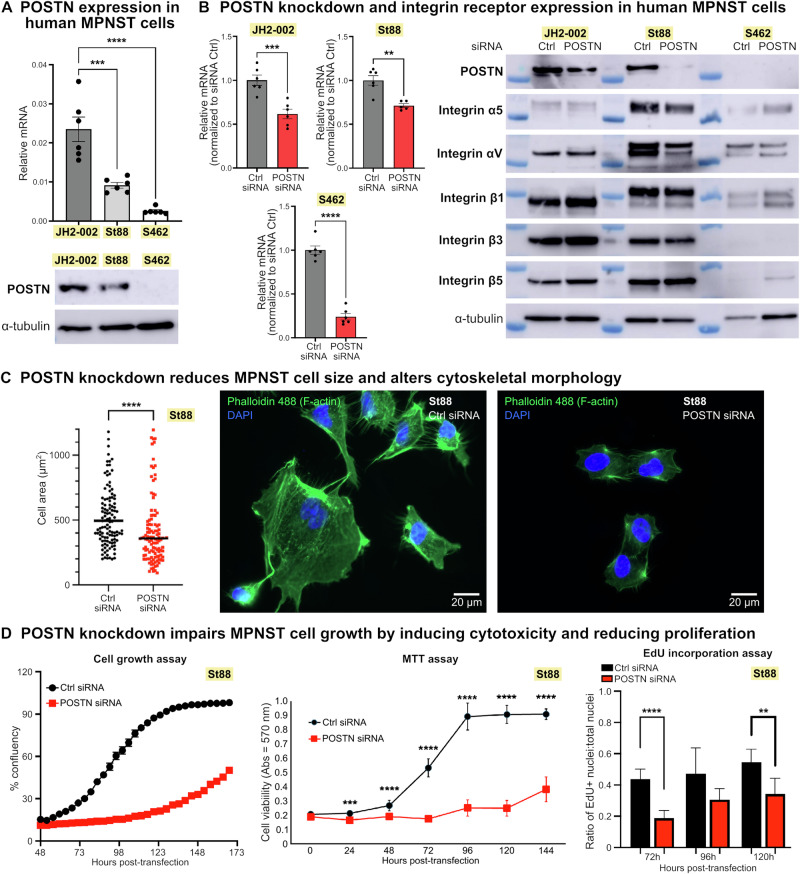
Periostin (POSTN) knockdown profoundly impacts MPNST cell morphology and growth. **A** The human MPNST cell lines JH2-002, St88, and S462 express POSTN, with variability in expression level between cell lines (see also Fig. S9A, S8A for POSTN expression in S462 cells). **B** siRNA-mediated knockdown of POSTN in MPNST cells reduces its expression at both mRNA and protein levels, while expression of integrin receptor subunits is largely unaffected (see also Fig. S9B, S8B for POSTN expression in S462 cells). **C** POSTN knockdown significantly reduces MPNST cell size, as quantified by cytoplasmic area, and drastically alters cytoskeletal architecture (*n* = 120 cells per condition, imaged after 48 h incubation with control (Ctrl) or POSTN siRNA). Data for St88 cells are shown. **D** POSTN knockdown impairs MPNST cell growth by significantly reducing both cell viability and proliferative capacity. Data for St88 cells are shown. Significance, *p ≤ 0.05, **p ≤ 0.005, ***p ≤ 0.0005, and ****p ≤ 0.0001. See Fig. S8A, S9A for experimental timeline.

After determining that POSTN is highly expressed in human MPNST samples, we tested the functional effects of POSTN deficiency in MPNST cells (Fig. S8A). We detected POSTN expression in three human MPSNT cell lines (JH-002, St88, and S462) (Figs. 6A and S9A) and used an siRNA pool against POSTN to knock down its expression in each cell line (Figs. 6B, S8B, and S9B). POSTN signaling is mediated by integrin receptors, and integrins α5β1, αVβ3, and αVβ5 function as receptors for POSTN in other human cancer types [43, 44]. We found that all three MPNST cell lines express multiple integrin α and β subunits, with their expression largely unaffected by POSTN knockdown (Figs. 6B and S10).

Upon POSTN knockdown in MPNST cells we observed a significant reduction in cytoplasmic area accompanied by marked alterations in cytoskeletal morphology (Figs. 6C and S8C). Time-course analyses of cell growth after POSTN knockdown suggested severe growth retardation in all three MPNST cell lines based on percent confluency (Figs. 6D and S8D). Since the reduced cell area observed in MPNST cells with POSTN knockdown could impact assessments of confluency, we analyzed cell growth by MTT and EdU incorporation assays. These assays demonstrated growth impairment after POSTN knockdown that was attributable to both increased cell death as well as a decreased proportion of actively proliferating cells (Figs. 6D and S8D), suggesting that POSTN deficiency may have both cytotoxic and cytostatic effects in MPNST cells.

## Discussion

The microenvironment is increasingly recognized as a significant contributor to cancer initiation, wherein carcinogenesis may require both a transformed clone and a synchronously altered local microenvironment that supports the survival of cancer-initiating cells [1, 2]. However, characteristics that define this precancerous or premalignant niche are not well understood.

Zebrafish (*Danio rerio*) provides an excellent complementary animal model for analyzing microenvironmental cell-cancer cell interactions [45]. Zebrafish have been used previously to identify tumor-promoting interactions between preneoplastic or early cancer cells and surrounding cells [21, 46, 47]. Although these investigations preserved the natural pathophysiology of tumor initiation and progression in an in vivo setting, they were conducted with larval zebrafish and thus may not fully capture intercellular interactions relevant to carcinogenesis in adult animals.

We built upon these prior studies using a zebrafish model for MPNST that enabled global characterization of the gene expression profiles for precancerous and cancer cellular microenvironments. The ONP is a cancer predilection site in this model [8–10] and was the focus of these analyses since ocular tissues are circumscribed by the infraorbital bones [17, 18] and can be collected in a consistent manner. MPNST in our zebrafish model results from combined inherited mutations in *brca2* and *tp53* [3, 10], which differs from the most common genetic contributors to MPNSTs in humans [11, 48]. These differences are considerations in interpreting our results. However, human MPNSTs do acquire somatic TP53 mutations [11, 13] and have been found to exhibit evidence of “BRCAness” [12, 49, 50].

Our analyses of the precancerous and cancer microenvironment in the zebrafish MPNST model suggest broad activation of multiple immune- and inflammation-associated pathways, which may indicate that local inflammation promotes MPNST initiation and progression. Similarly, in mouse models, neurofibroma is promoted by an inflammatory microenvironment [51]. Inflammation has been suggested broadly as a major contributor to formation of the precancerous niche and a driver of cancer initiation and progression [1, 2], which is supported by studies in mice [52–55] and zebrafish [21, 46, 47]. As investigations into the role for inflammation in MPNST are limited, further studies are required to define this relationship.

We identified macrophages as potential contributors to microenvironmental phenotypes in zebrafish MPNST. Previous studies have implicated macrophages in promoting the growth of both neurofibroma and MPNST [56–59], although macrophage enrichment is significantly impacted by mouse strain in MPNST mouse models [60]. Our analysis suggests the presence of both M1- and M2-polarized macrophages in precancerous and cancer microenvironments, similar to a neurofibroma mouse model [61]. While the presence of presumptive macrophages at cancer margins in our model suggests a role in invasive behavior, further studies are required to assess macrophage-MPNST interactions.

In a cross-species comparative analysis, we found most differentially expressed genes in MPNST versus benign counterparts in both humans and zebrafish showed concordant expression changes, including multiple up-regulated genes independently identified as contributors to MPNST progression [40–42, 62]. We confirmed expression of two additional upregulated gene candidates, POSTN and CTHRC1, that have not been previously assessed in human MPNST but are known contributors to progression in other human cancer types [63–66]. Although we did not observe clear differences in POSTN expression in benign versus malignant human samples, sample size and tumor heterogeneity were likely confounders (as suggested by frequent differences in expression scores in duplicate cores (Fig. S6B)). We also confirmed expression of zebrafish orthologues for POSTN in zebrafish MPNSTs. While overall expression was low, possibly due to the age of these specimens, we noted a predilection for POSTN expression at cancer cell-normal tissue interfaces.

We performed preliminary functional validation of POSTN as a contributor to human MPNST progression. Our in vitro studies confirmed expression of POSTN in three human MPNST cell lines, as well as expression of integrin receptors that mediate POSTN signaling in other human cancer types [43, 44]. We showed that POSTN knockdown altered cellular morphology and reduced cell growth through both increased cytotoxicity and decreased proliferation in all three MPNST cell lines. We note some variability across cells lines in POSTN expression level and responses to POSTN knockdown (Figs. 6 and S8). The impact of POSTN knockdown was relatively less severe in the cell line with the lowest levels of expression of POSTN and integrin receptor subunits (S462 cells), suggesting that these cells may be comparatively less reliant on POSTN signaling. Irrespective of these individual cell line variations, our data demonstrate that POSTN knockdown profoundly impacts MPNST cell growth and survival. These data support further investigation of POSTN as a therapeutic target in MPNST and demonstrate the utility of human-zebrafish comparative genomics analyses in identifying conserved genetic contributors to cancer.

Limitations to this study include the use of RNA expression profiling to define precancerous and cancer microenvironments, which may not correlate to protein expression. Also, many proteins require post-translational modifications for functionality that are not captured by RNA-seq analysis. Finally, bioinformatics analysis was facilitated by zebrafish-to-human orthologue mapping, with duplicated zebrafish genes summed to the level of the most significantly differentially expressed orthologue. While this conversion enabled comparative gene expression and pathway analyses, potential differences in the contributions of specific zebrafish orthologues to specific phenotypes are not captured.

The tumor microenvironment for soft tissue sarcomas such as MPNST is relatively uncharacterized, and little is known about microenvironmental factors that may promote or repress sarcomagenesis. The prognosis remains poor for MPNST patients due to aggressive infiltrative growth, frequent metastasis, and limited response to conventional or targeted therapies. The current study provides new insight into candidate microenvironmental factors in sarcomagenesis and highlights potential contributors to MPNST initiation and progression for future study.

## Materials and methods

### In vivo animal studies

Experiments were performed with adult zebrafish and included precancerous and cancerous cohorts (*tg(sox10:RFP);brca2*^*hg5/hg5*^*;tp53*^*zdf1/zdf1*^) and a control cohort (*tg:sox10:RFP*) [19]. Specific details for the study population are in Table 1. Ocular tumor specimens used for protein isolation were derived from additional *tg(sox10:RFP);brca2*^*hg5/hg5*^*;tp53*^*zdf1/zdf1*^ zebrafish upon tumor development. All animal studies were approved by the Institutional Animal Care and Use Committee, North Carolina State University, Raleigh, NC, and by the Institutional Care and Use Committee, The Ohio State University, Columbus, OH. Animal studies were performed in accordance with approved protocols and complied with ARRIVE guidelines.

### Bioinformatics analysis

The quality of sequenced reads were assessed using FastQC, and good-quality reads were aligned to the Zebrafish reference genome (GRCz10 version 87) downloaded from the Ensemble database using the STAR aligner [67]. Unique Ensembl gene counts were tabulated using the HTSeq python package [68] for each sample. RNA-seq data has been deposited at GEO and are publicly available as of the date of publication (GEO: GSE198220).

### Differential gene expression analysis

Normalization of raw counts and differentially expressed gene lists were generated with the DESeq2 [69] package in *R 4.0* after using the *org.Dr.eg.db* package (Marc Carlson (2021). org.Dr.eg.db: Genome wide annotation for Zebrafish) to map Ensembl gene IDs to gene name. Counts were summed to unique gene symbol prior to DESeq normalization and analysis. Genes with less than one count in one half of the sample space were removed. Normalized counts, log_2_ fold change in gene expression, and adjusted *p*-values [70] were calculated for each treatment comparison using DESeq2. For some downstream analyses, a “humanized” gene list was generated for each comparison by merging identified zebrafish gene names with human orthologous gene names (Zebrafish Information Network (ZFIN) “Human and Zebrafish Orthology” dataset, https://zfin.org/downloads). Where human gene symbols mapped to duplicated zebrafish genes, expression data for the zebrafish orthologue with the smallest associated adjusted *p*-value was retained for subsequent analysis in IPA.

### Cross-species comparative genomics analysis

Gene lists from a previous study reporting differential gene expression in human MPNST and ANNUBP (atypical neurofibromatosis neoplasm of unidentified biologic potential [39]) versus neurofibroma [38] were compared to the “humanized” gene lists generated as described above for the following comparisons: zebrafish cancer cells versus potential precancerous cells; zebrafish cancer ME cells versus precancerous ME cells. For this analysis, differentially expressed genes in zebrafish datasets were identified by the following criteria: abs(log2 fold change) >1.5 and adjusted *p* < 0.05. These parameters are equivalent to the parameters used to identify differentially expressed genes in the human study [38].

### Analyses with human patient samples and cell lines

Human specimens used in experiments included anonymized tumor samples from human MPNST patients; a commercially available tissue microarray; and MPNST cell lines sNF96.2, JH2-002, St88, and S462. IRB approval was not required for the use of these specimens. See Supplemental Materials and Methods for experimental details.

## Supplementary information

Combined supplementary files

Table S1

Table S2

Table S3

## Data Availability

The datasets generated during and/or analyzed during the current study have been deposited in GEO (https://www.ncbi.nlm.nih.gov/geo/) and are publicly available as of the date of publication (GEO: GSE198220).
