# Supplementary material for: Profiling the cancer-prone microenvironment in a zebrafish model for MPNST
Source: Oncogene. 2024 Nov 7;44(3):179–91. doi: 10.1038/s41388-024-03210-1 (PMC11725499; doi:10.1038/s41388-024-03210-1)

Title: Profiling the cancer-prone microenvironment in a zebrafish model for MPNST

Supplementary Materials and Methods

Study population. Experiments were performed with adult zebrafish. Zebrafish used for precancerous and cancerous cohorts (*tg(sox10:RFP);brca2*$^{hg5/hg5}$*;tp53*$^{zdf1/zdf1}$) were from separate clutches derived from the same parents. The control cohort (*tg:sox10:RFP*)[1] was maintained as a separate line. For precancerous and control cohorts, zebrafish were randomly selected from their respective genotypic groups in approximately equal numbers of males and females. Zebrafish for the cancer cohort were monitored for ONP tumor development and collected upon tumor development. Specific details for age, sex, and animal numbers for the study population are in **Table 1**. Ocular tumor specimens used for protein isolation were derived from *tg(sox10:RFP);brca2*$^{hg5/hg5}$*;tp53*$^{zdf1/zdf1}$ zebrafish upon tumor development. Investigators were not blinded to the genotype of animals used in this study. All animal studies were approved by the Institutional Animal Care and Use Committee, North Carolina State University, Raleigh, NC and by the Institutional Care and Use Committee, The Ohio State University, Columbus, OH. Animal studies were performed in accordance with approved protocols and complied with ARRIVE guidelines.

Zebrafish husbandry. Zebrafish used in this study were raised on a Z-Hab Duo recirculating aquaculture system (Pentair, Apopka, FL, USA) and maintained on a 14-hour light/10-hour dark cycle. The zebrafish colony undergoes routine sentinel testing and is negative for known zebrafish pathogens. Live adult zebrafish were genotyped for the *brca2*^{hg5} mutation[2] at three months of age by sequencing and were maintained as homozygous mutants for the *tp53*^{zdf1} mutation[3]. Carriers of the *sox10:RFP* transgene were identified in each generation by fluorescence stereomicroscopy. All zebrafish collected for analysis were euthanized with Tricaine

methanesulfonate (300 mg/L) in system water buffered with Sodium Bicarbonate to a pH of ~7.0 or in an ice water slurry per our IACUC-approved Animal Study Protocol.

Tissue and fluorescence-activated cell sorting (FACS). The method for tissue dissociation was modified from a previously published protocol[4]. Tissues from the optic nerve pathway (**Table 1**) were dissected, placed in L-15 medium, and minced. Tissues were enzymatically dissociated in 0.05% Trypsin/0.5 mM EDTA in sterile water at 28ºC for 45 minutes with gentle pipetting every 15 minutes. An equal volume of trypsin inhibitor solution (0.52 mg/ml Trypsin inhibitor, type III-O, 3 mg/ml Bovine Serum Albumin, fraction V, and 0.004% DNase in L-15 medium) was added for trypsin inactivation. Samples were triturated three times with a 25-guage needle, filtered with a 35 um filter, and resuspended in cold FACS buffer (2% Fetal Bovine Serum in 1X Hank's Buffered Saline Solution). Cell suspensions were stained with SYTOX Green Dead Stain (Invitrogen) and analyzed with a MoFlo XDP Sorter (University of North Carolina Flow Cytometry Core; Beckman Coulter, Brea, CA, USA) (**Fig. S1**). The MoFlo XDP Sorter was maintained and calibrated daily according to the amnufacturer's recommendation. Maximal numbers of RFP-positive and RFP-negative populations were collected into L-15 medium, pelleted, resuspended in QIAZol reagent (Qiagen, Germantown, MD, USA), and frozen on dry ice.

RNA isolation, library preparation, and RNA sequencing. Total RNA from zebrafish specimens was isolated with an miRNeasy Micro Kit (Qiagen, Germantown, MD, USA) according to the manufacturer's protocol. RNA integrity, purity, and concentration, Illumina RNA library construction, and sequencing were performed using total RNA (NC State University Genomic Sciences Laboratory, Raleigh, NC) as previously described[5]. RNA-seq data has been deposited at GEO and are publicly available as of the date of publication (GEO: GSE198220).

Immunohistochemical analyses of zebrafish tissues. Fifteen ONP cancers from *brca2*$^{hg5/hg5}$;*tp53*$^{zdf1/zdf1}$ zebrafish that were collected and reported in a previous study[6] were used for immunohistochemical analyses. Immunohistochemistry on unstained paraffin sections from these specimens was performed as previously described[7, 8]. Zebrafish spleen and kidney were used as positive controls for lcp1 and mpx1 expression, respectively (**Fig. S1D,E**). Normal brain and eye provided internal positive controls for sox10 expression in cancer-bearing zebrafish (**Fig. 1B, Fig. S3P**). Sections incubated without primary antibodies were used as negative controls (**Fig. S1H, Fig. S3Q**). Details on antibodies used are in **Table S4**.

Immunohistochemical analyses of human tissues. A commercially available tissue microarray (TMA) composed of duplicate core biopsies from human peripheral nerve tumor tissues and commercially available sections of normal human colon were used for immunohistochemical analyses (#SO1001b, TissueArray.com LLC, Derwood, MD, USA). Due to the use of outdated nomenclature in pathologic diagnoses provided by the manufacturer for some core specimens, samples were designated as benign or malignant and are reported as such. The TMA included 22 cores from malignant tumors and 20 cores from benign tumors. Immunohistochemistry was performed using a Bond Rxm autostainer (Leica Biosystems, Wetzlar, Germany) according to the manufacturer's protocol with the following specifications: 15 minute antigen retrieval, pH 6.0; 15 minute incubation with primary antibody solution. Sections of normal human colon were processed with and without primary antibody as within-run positive and negative assay controls, respectively (**Fig. S6C**). A semiquantitative scale was used to assess the distribution and intensity of antibody labeling in each core biopsy (**Fig. S6B**). Scoring was performed independently by visual estimate by two veterinary pathologists (OMP, HRS) with final score determined in consensus. Each core was scored individually for distribution and intensity of labeling and scores were averaged for each pair of duplicates (**Fig S6B**). Details on antibodies used are in **Table S4**.

RNA in situ hybridization analyses of zebrafish tissues. RNA in situ hybridization was performed using the RNAscope system (Advanced Cell Diagnostics (ACD), Newark, CA, USA) using custom RNA probes for zebrafish *postna* and *postnb* designed by the manufacturer. The RNAscope assay was performed on 5µm paraformaldehyde-fixed, paraffin-embedded tissue sections using the 2.5 HD Duplex kit (ACD) according to the manufacturer's instructions with the following modifications: slides were incubated with Target Retrieval and Protease Plus reagents for 30 minutes and 30 minutes, respectively. A universal 2-plex negative control with probes targeting the *dapB* gene (ACD) was used as a negative control. 2.5% Gills Hematoxylin was used as a counterstain and slides were mounted using Vectamount medium.

Tissue and slide imaging*.* Zebrafish tissues used for IHC analyses were imaged with a Lumar V12 stereomicroscope and Axiovision software (Zeiss, Baden-Wurttemberg, Germany). Digital image files from histologic slides of zebrafish specimens were created by imaging with an Olympus BX43 brightfield microscope with DP26 camera and Olympus cellSens Imaging Software or by scanning with an Olympus VS200 Research Slide Scanner with Hamamatsu Orca Fusion camera and Olympus OlyVIAViewer software (Olympus, Center Valley, PA, USA). Digital image files from histologic slides of human specimens were created as whole slide images using an AT2 digital slide scanner (Leica Biosystems). Zebrafish tissues used for RNA ISH analyses were scanned at 63x magnification on an Aperio VERSA 8 (Leica Biosystems). Digital images were minimally and globally processed with the GNU Image Manipulation Program for white balance, contrast, and exposure.

Human cell lines. sNF96.2 cells were cultured at 37ºC under 5% CO_2 in DMEM-HG (Gibco) with 10% (vol/vol) fetal calf serum (GeminiBio). sNF96.2 cells were obtained from the American Type Culture Collection (Cat. #CRL-2884) and were not authenticated. JH2-002, St88 and S462 cells were kindly provided by Drs. Jack Shern and Bega Murray (NCI CCR Pediatric Oncology Branch)

and were authenticated by STR profiling by the providers in December 2022. JH2-002, St88 and S462 cells were cultured in RPMI (Gibco) with 10% (vol/vol) fetal calf serum (GeminiBio) and 1% Pen/Strep (Gibco). All cell lines were were routinely tested for mycoplasma contamination using a previously validated16S rRNA-based mycoplasma group-specific PCR assay[9] or with the MycoAlert Mycoplasma Detection Kit (Lonza Bioscience, Walkersville, MD, USA).

POSTN knockdown experiments. For siRNA experiments, cells were plated one day prior to transfection. Cells were transfected with 15nM siRNA targeting POSTN (ON-TARGETplus Human POSTN (10631) siRNA – SMARTpool; Cat. #L-020118-00-0005; Horizon Discovery, Cambridge, UK) or control siRNA (ON-TARGETplus Non-targeting Pool; Cat. #D-001810-10-05; Horizon) using Lipofectamine (Invitrogen) and incubated with siRNA/Lipofectamine for 48 hours. After 48 hours, the medium was replaced with fresh complete medium and cells were used for experimental assays as show in **Fig. S8A**.

RNA extraction and quantitative RT-PCR analyses of human cell line samples. Total RNA was isolated from human cell lines using Trizol (Invitrogen) according to the manufacturer's protocol. RNA quantity and quality were assessed by spectrophotometry (NanoDrop ONE, Waltham, MA, USA). 2 ug of total RNA was reverse-transcribed using High Capacity cDNA Reverse Transcription Kit (Cat.# 4368814; Applied Biosystems) according to the manufacturer's protocol. qRT-PCR analysis was performed with duplicate samples using SYBR green fluroscent dye (Cat.# AZ-2350; Azura Genomics) and quantified with the ABI Prism 7900 sequence-detection system (Applied Biosystems). Periostin (POSTN) gene expression was normalized to the expression of the housekeeping gene Actin. When comparing mRNA expression between cell lines treated with control or POSTN siRNA, gene expression was normalized to the cell line treated with control siRNA.

Gene	Primer	Sequence
Actin	sense	5'-GAG CTA CGA GCT GCC TGA CG-3'
	antisense	5'-GTA GTT TCG TGG ATG CCA CAG-3'
POSTN	sense	5'-CAA CGC AGC GCT ATT CTG AC-3'
	antisense	5'-CCA AGT TGT CCC AAG CCT CA-3'

Protein isolation from zebrafish and human tissues. Ocular tumors from three *tg(sox10:RFP);brca2*$^{hg5/hg5}$*;tp53*$^{zdf1/zdf1}$ were collected for protein isolation. Whole-cell lysates from zebrafish ocular tumors or cultured cells were prepared by lysing cells in RIPA buffer (50 mM Tris pH 7.4; 150 mM NaCl; 1mM EDTA; 1% Triton-X; 0.1% SDS) with complete protease inhibitors (Roche, Indianapolis, IN, USA) and clarified by centrifugation. Tissue samples were homogenized on ice with a motor-driven pestle during lysis. Anonymized frozen tumor samples derived from human patients diagnosed with MPNST were acquired from the Ohio State University Comprehensive Cancer Center Biospecimen Services Shared Resource. Frozen samples were placed in RIPA buffer with complete protease inhibitors (Roche) and whole-cell lysates were prepared by homogenizing tissues with a Precellys 24 tissue homogenizer (Bertin Technologies, Montigny-le-Bretonneux, France) according to the manufacturer's protocol. Protein quantification was performed by Bradford assay or by spectrophotometry (NanoDrop ONE; Abs = 280nm).

Western blotting. Whole-cell lysates were separated by SDS-polyacrylamide gel electrophoresis in NuPAGE Bis-Tris precast gels in MOPS running buffer and electroblotted onto 0.45 μm PVDF membranes using a wet transfer system. Membranes were incubated for one hour at room temperature in in 5% w/v bovine serum albumin (BSA) in Tris-buffered saline-0.1% Tween-20 (TBST) or in 5% w/v non-fat dry milk in PBS-0.05% Tween-20 (PBST) and then incubated overnight at 4ºC with the primary antibody diluted in the same buffer used for blocking. Membranes were washed four times in TBST or PBST and then incubated with the secondary antibody in blocking buffer for one hour at room temperature. Membranes were rewashed four

times in TBST or PBST, incubated in a 1:3 mixture of ECL Pico WB Substrate and ECL Femto Maximum Sensitivity substrate or in ECL Femto alone, and imaged with a ChemiDoc imager (Bio-Rad Laboratories, Hercules, CA, USA) or Amersham Imager 600 (Cytiva, Marlborough, MA, USA) using the chemiluminescence imaging function. For some western blots, sequential detection of primary antibodies was performed on the membranes. In these cases, the membrane was processed as described above, imaged, and subsequently washed before re-processing with the next primary antibody. Details on antibodies used are in **Table S4**.

Cell imaging and area measurement. Cells were plated at a concentration of 2x10^4 cells/well onto 18 mm diameter-coverslips and transfected with 15 nM POSTN siRNA or control siRNA as described above. 48 hours after transfection, cells were fixed with 4% PFA in PBS for 10 minutes at room temperature, washed in 1X PBS, and incubated with 1X Alexa Fluor™488 Phalloidin (Cat. # A12379; Invitrogen) in PBS for 10 min at RT. Cells were washed once in 1X PBS, once in dH_2O, and the coverslips were gently mounted on slides using a drop of VECTASHIELD® Vibrance antifade mounting medium with DAPI (Cat. # H-1800; Vector Laboratories). Images were acquired with a ZEISS Axio Imager.M2 upright microscope equipped with 5-megapixel monochrome CMOS Axiocam 705 camera and processed with ZEN microscopy software. Single cell area in μm^2 was measured in ImageJ by manually tracing the perimeter of 120 individual cells per condition and proceeding to the analyze>measure>area Image-J function, after training Fiji with the scalebar of ZEN software. The numbers of cells selected for analysis was based on a review of literature reporting similar analytical studies in cultured cells. Samples were not randomized, and researchers were not blinded during measurements and in experimental condition assignment.

Cell proliferation assays. Cells were plated in triplicate in 24-well plates at a concentration of 4x10^4 cells/well (JH2-002, St88) or 2x10^4 cells/well (S462) and treated with 15 nM POSTN or

control siRNA as described above. After completion of transfection and media change, cells were evaluated using an Incucyte S3 Live Cell Analysis System (Sartorius, Ann Arbor, MI) using 4h interval scans for up to 5 days. Phase contrast images series were analyzed using Incucyte software to measure the area in the well covered by cells and data were processed using GraphPad Prism 10 (GraphPad Software, San Diego, CA, USA).

MTT cell viability/cytotoxicity assay. Cells were plated in 96 well plates at a concentration of 7,000 cells/well (JH2-002 and St88) or 5,000 cells/well (S462), with 16 replicates per condition, and treated with 15 nM POSTN or control siRNA as described above. The day of the measurement, 10 µL of MTT solution (5 mg/mL dissolved in sterile water, Invitrogen Cat.# M6494) was added to each well for each plate at collection timepoints indicated in **Fig. S8A**. Cells were incubated with MTT for 2-3 hours at 37°C, 5% CO_2. After MTT incubation, the medium was gently removed, and 100 µL of pure DMSO was added to each well to dissolve the formazan crystals by gentle pipetting, to prevent foaming. After a 15-minute incubation at 37°C, absorbance was measured at 570 nm using Infinite® 200 PRO Tecan microplate reader with i-control™ software.

EdU cell proliferation assay. Cells were plated in 24-well plates at a concentration of $2x10^4$ cells/well in triplicate and treated with POSTN or control siRNA as described above. After completion of transfection and media change, cells were labeled with EdU according to the manufacturer's protocol (Click-iT™ EdU Cell Proliferation Kit, Cat.# C10337; Invitrogen). After 2 hours incubation in 10 µM EdU, cells were fixed in 4% PFA at room temperature for 15 minutes and permeabilized with 0.1% Triton X-100 in PBS for 20 minutes at room temperature. The incorporated EdU was labeled with the Click-iT reaction cocktail containing Alexa Fluor 488 azide for 30 min at room temperature, protected from light. All cells were counterstained with Incucyte® Nuclight Rapid Red Dye (Sartorius 4717). The green and red fluorescent images of labeled cells were acquired using Incucyte S3 (Sartorius) at 10x magnification with exposure time 0.3 second

(green) and 0.4 second (red). The number of EdU-positive (green) cells and total cells (red) were counted using the Incucyte analysis tool. The percentage of proliferating cells was represented by the ratio of green cells to red cells.

Statistical analyses for in vitro experiments. GraphPad Prism version 10.2.3 for Mac (GraphPad Software, San Diego, CA, USA) was used for graphical representation and statistical analysis. The significance of differences among groups was evaluated with two-tailed Student's t-test for comparisons between two groups, based on normality distribution checked by the Shapiro-Wilk test. For datasets that did not follow a normal distribution, non-parametric tests like the Mann-Whitney test for two groups comparisons and One Way ANOVA for three groups were applied. Statistical significance was considered at a p-value ≤ 0.05, with significance indicated as *$p \leq 0.05$, **$p \leq 0.005$, ***$p \leq 0.0005$, and ****$p \leq 0.0001$. Data are shown as mean or median (where specified) ± standard deviation (SD).

Ingenuity Pathways Analysis (IPA). Data were analyzed through the use of IPA[10] (QIAGEN Inc.,
https://www.qiagenbioinformatics.com/products/ingenuitypathway-analysis; mapping database version Q2 2020 and gene model source version Hg38/mm38 and Hg19/mm10 from UCSC). IPA core analysis was performed with RNAseq data mapped to human gene names (generated as described above). Data input settings for core analysis were as follows: reference set = user dataset; $\log_2$ fold change cutoff = -1.0 to 1.0; adjusted p-value < 0.05. This allows for discovery while limiting false discovery to 5%. Top pathways were identified by $-\log_{10}$(enrichment p-value) and activation z-score, where applicable, and were selected based on $-\log_{10}$(enrichment p-value) ≥ 1.3 and activation z-score (activated pathways, z-score ≥ 1.0; inhibited pathways, z-score ≤ -1.0). The complete list of canonical pathways identified is in **Table S1**. The complete list of predicted upstream regulators is in **Table S2**.

The significance of the associations between data sets and canonical pathways was measured as previously described[10]. Upstream regulators identified by IPA core analysis were filtered by z-score, log2 fold change, p-value, and predicted activation state. Upstream regulators predicted to result in pathway activation were identified by z-score > 1.0, log2 fold change > 1.0, and p-value < 0.05. Upstream regulators predicted to result in pathway inhibition were identified by z-score < -1.0, log2 fold change < 1.0, and p-value < 0.05.

GSEA analysis. Gene sets for precancerous versus control microenvironments, cancer versus control microenvironments, and cancer versus precancerous microenvironments were uploaded to GSEA 4.3.3. Analysis was performed with the GSEA Preranked Tool using Hallmark Gene Sets (H) with 1000 permutations and without gene set collapse. A false discovery rate (FDR) of <25% was applied for statistical significance as directed in the GSEA User Guide. The complete list of Hallmark gene sets with corresponding normalized enrichment score and FDR q-values is in **Table S3**.

References

1 Blasky AJ, Pan L, Moens CB, Appel B. Pard3 regulates contact between neural crest cells and the timing of Schwann cell differentiation but is not essential for neural crest migration or myelination. *Dev Dyn* 2014; 243: 1511-1523.

2 Shive HR, West RR, Embree LJ, Azuma M, Sood R, Liu P, Hickstein DD. brca2 in zebrafish ovarian development, spermatogenesis, and tumorigenesis. *Proc Natl Acad Sci U S A* 2010; 107: 19350-19355.

3 Berghmans S, Murphey RD, Wienholds E, Neuberg D, Kutok JL, Fletcher CD *et al*. tp53 mutant zebrafish develop malignant peripheral nerve sheath tumors. *Proc Natl Acad Sci U S A* 2005; 102: 407-412.

4 Bogler O. Isolation and purification of primary oligodendrocyte precursors. *Curr Protoc Neurosci* 2001; Chapter 3: Unit 3 4.

5 Arambula SE, Jima D, Patisaul HB. Prenatal bisphenol A (BPA) exposure alters the transcriptome of the neonate rat amygdala in a sex-specific manner: a CLARITY-BPA consortium study. *Neurotoxicology* 2018; 65: 207-220.

6 Mensah L, Ferguson JL, Shive HR. Genotypic and Phenotypic Variables Affect Meiotic Cell Cycle Progression, Tumor Ploidy, and Cancer-Associated Mortality in a brca2-Mutant Zebrafish Model. *J Oncol* 2019; 2019: 9218251.

7 Kouprianov VA, Selmek AA, Ferguson JL, Mo X, Shive HR. brca2-mutant zebrafish exhibit context- and tissue-dependent alterations in cell phenotypes and response to injury. *Sci Rep* 2022; 12: 883.

8 White LA, Sexton JM, Shive HR. Histologic and Immunohistochemical Analyses of Soft Tissue Sarcomas From brca2-Mutant/ tp53-Mutant Zebrafish Are Consistent With Neural Crest (Schwann Cell) Origin. *Vet Pathol* 2017; 54: 320-327.

9 van Kuppeveld FJ, Johansson KE, Galama JM, Kissing J, Bolske G, van der Logt JT, Melchers WJ. Detection of mycoplasma contamination in cell cultures by a mycoplasma group-specific PCR. *Appl Environ Microbiol* 1994; 60: 149-152.

10 Kramer A, Green J, Pollard J, Jr., Tugendreich S. Causal analysis approaches in Ingenuity Pathway Analysis. *Bioinformatics* 2014; 30: 523-530.

11 Thisse B, Pflumio, S., Fürthauer, M., Loppin, B., Heyer, V., Degrave, A., Woehl, R., Lux, A., Steffan, T., Charbonnier, X.Q. and Thisse, C. Expression of the zebrafish genome during embryogenesis (NIH R01 RR15402). ZFIN Direct Data Submission., 2001 edn. The Zebrafish Information Network, 2001.

12 Thisse B, Thisse, C. Fast Release Clones: A High Throughput Expression Analysis. ZFIN Direct Data Submission. The Zebrafish Information Network, 2004.

13 Kudo H, Amizuka N, Araki K, Inohaya K, Kudo A. Zebrafish periostin is required for the adhesion of muscle fiber bundles to the myoseptum and for the differentiation of muscle fibers. *Dev Biol* 2004; 267: 473-487.

Supplementary Figure Legends

Figure S1. Zebrafish MPNST model characterization, gating strategy for fluorescence-activated cell sorting, and immunohistochemical analyses. A, Zebrafish ONP cancers exhibit AKT and ERK1/2 activation. sNF96.2, human MPNST cell line. OT1, OT2, and OT3, ONP cancers from *tg(sox10:RFP);brca2* $^{hg5/hg5}$*;tp53*$^{zdf1/zdf1}$ zebrafish. Cyclophilin B (CYPB) expression was used as a loading control and this antibody did not detect zebrafish cypb. The membrane was cut into sections for western blotting and reassembled for imaging. B-C, A similar gating strategy was applied to control (not shown), precancerous (B), and cancer (C) specimens. Forward and side scatter were used to eliminate debris and doublets. GFP expression (SYTOX Green Dead Stain; Invitrogen) was used to exclude dead cells. Single live cells were subsequently sorted into RFP-positive and RFP-negative populations. The final panels are also shown in **Fig. 1F**. D, lcp1 expression in zebrafish spleen (purple chromagen). E, mpx1 expression in zebrafish kidney (purple chromagen). F, Lcp1-positive macrophages are present throughout the tumor and are primarily concentrated along peripheral margins. G, Numerous mpx1-positive neutrophils are distributed throughout the tumor, with limited overlap of lcp1 and mpx1 positivity. H, Negative control incubated without primary antibody. Box 1 shows areas of melanin pigmentation.

Figure S2. Site for tissue collection and experimental design. A, Schematic of the zebrafish head indicating the site for tissue collection from the orbit and major tissue types present in this region. ON, optic nerve. Note that the bones comprising the orbit and skull are not shown as individual structures for simplicity. B and C, Histologic sections from a wild type zebrafish demonstrating the tissues present in the orbit and the bone margin surrounding the orbit (blue arrows). Panels 1-3 in C are higher magnification images of the numbered regions in B. R, retina; M, muscle; CR, choroid rete; A, adipose; B, brain; L, lens.

Figure S3. ONP cancers from *brca2* $^{hg5/hg5}$*;tp53*$^{zdf1/zdf1}$ zebrafish exhibit widespread sox10 expression. A-O, 15 ONP cancers from *brca2* $^{hg5/hg5}$*;tp53*$^{zdf1/zdf1}$ that were described in a previous study[7] were analyzed for sox10 expression (brown chromogen) by immunohistochemistry. The boxed region in panel D is shown at higher magnification in **Fig. 1B**. P, Boxed region in panel O showing sox10-positive cells in brain (internal positive control). Q, Negative control incubated without primary antibody.

Figure S4. Quality control analysis of RNAseq data. A, Read counts for individual samples. B, Cluster dendrogram of individual experimental replicates for each analyzed sample.

Figure S5. Enriched canonical pathways that are shared across multiple comparisons. The pathways shown were identified as statistically significantly affected (see **Methods** for details). A, Commonly enriched canonical pathways without predicted directional activity. B, Commonly enriched pathways with predicted pathway activation. C, Commonly enriched pathways with predicted pathway inhibition. PC ME, precancerous microenvironment; C ME, cancer microenvironment; CTL ME, control microenvironment.

Figure S6. Candidate gene testing in human MPNST samples. A, Full Western blots for expression of CTHRC1 and POSTN in MPNST samples from human patients. Blots were probed for detection of CTHRC1 or POSTN and imaged, then washed and re-probed for detection of α-tubulin. B, POSTN expression (brown chromogen) in a tissue microarray comprised of human peripheral nerve tumor tissue. Solid outline, malignant tumor specimens (A1-E4); dashed line, benign tumor specimens (E5-I4). Representative examples of various scoring outcomes in individual core biopsies are shown. Graphical representations of scoring outcomes for distribution and intensity show the scores for individual core biopsies, aligned in duplicate pairs. Red lines

connect unequal scores for duplicate pairs. C, POSTN expression (brown chromogen) in normal human colon. The negative control was incubated with secondary antibody only.

Figure S7. Expression of *postna* and *postnb* in zebrafish MPNSTs. A, Validation of RNA probes for *postna* (green chromogen) and *postnb* (red chromogen) in zebrafish embryos (4 days post-fertilization). The site of highest *postna* expression is the pharyngeal cartilage (black arrowheads), while highest sites of *postnb* expression include the skin (blue arrowheads) and intersomitic regions (yellow arrowheads). These expression patterns are consistent with data generated by large-scale analyses of gene expression in zebrafish embryonic and larval stages by whole-mount in situ hybridization[11, 12] and an analysis of periostin in myoseptum formation[13]. B, Moderate levels of *postna* and *postnb* expression in a zebrafish ONP cancer specimen. Black arrowheads, interface between optic nerve (ON) and tumor (T). C, Rare *postna* expression and low *postnb* expression in a zebrafish ONP cancer specimen. Black arrowheads, interface between epithelium (E) and tumor (T).

Figure S8. Periostin (POSTN) knockdown profoundly impacts MPNST cell morphology and growth. A, Timeline for experimental assays in MPNST cells treated with control (Ctrl) or POSTN siRNA. B, POSTN expression remains knocked down for up to 144 hours post-transfection in POSTN siRNA-treated cells compared to Ctrl siRNA-treated cells. C, POSTN knockdown significantly reduces MPNST cell size, as quantified by cytoplasmic area, and drastically alters cytoskeletal architecture (n = 120 cells per condition, imaged after 48 hours incubation with Ctrl or POSTN siRNA). Data for JH2-002 and S462 cell lines are shown. D, POSTN knockdown impairs MPNST cell growth by significantly reducing both cell viability and proliferative capacity. Data for JH2-002 and S462 cell lines are shown. Significance, *p ≤ 0.05, **p ≤ 0.005, ***p ≤ 0.0005, and ****p ≤ 0.0001.

Figure S9. Uncropped western blots for expression of periostin (POSTN) in human MPNST cell lines. A, POSTN expression in parental JH2-002, St88, and S462 cell lines. Blots were probed for detection of POSTN and imaged, then washed and re-probed for detection of α-tubulin. The blot used for POSTN is shown at two different exposures due to the lower expression level in S462 cells. B, POSTN expression 144 hours after transfection with POSTN siRNA or control siRNA in JH2-002, St88, and S462 cell lines.

Figure S10. Uncropped western blots for expression of periostin and integrin receptor subunits after POSTN knockdown in human MPNST cell lines. A, POSTN and integrin subunit expression in JH2-002, St88, and S462 cell lines after treatment with control siRNA or POSTN siRNA. Blots were sequentially probed for target detection with intervening washes between primary antibodies. Due to the lower abundance of POSTN and integrin β3 in S462 cells in comparison to JH-002 and St88 cells, these blots were re-imaged after covering the lanes containing lysates from JH-002 and St88 cells. Both full and partially covered blots are shown. B, Blots used for detection of POSTN and integrins receptor subunits in C were re-probed for detection of α-tubulin as a loading control.

Table S4. Antibodies used for immunohistochemistry and Western blotting experiments.

Antibody	Source	Identifier
anti-SOX10	GeneTex	Cat# GTX128374, RRID:AB_2885766
anti-Cyclophilin B	Cell Signaling Technology	Cat# 43603, RRID:AB_279924
anti-phospho-p44/42 MAPK(Erk1/2)(Thr202/Tyr204)	Cell Signaling Technology	Cat# 4370, RRID:AB_2315112
anti-p44/42 MAPK(Erk1/2)	Cell Signaling Technology	Cat# 4695, RRID:AB_390779
anti-phospho-AKT(Ser473)	Cell Signaling Technology	Cat# 4060, RRID:AB_2315049
anti-AKT (pan)	Cell Signaling Technology	Cat# 4691, RRID:AB_915783
anti-lcp1	GeneTex	Cat# GTX134697, RRID:AB_2887324
anti-mpx1	GeneTex	Cat# GTX128379, RRID:AB_2885768
anti-POSTN (used for analyses of human patient samples and tissue microarray)	GeneTex	Cat# GTX100602 RRID:AB_1951327
anti-POSTN (used for analyses of human MPNST cell lines)	Abcam	Cat# RM1074
Integrin antibody sampler kit	Cell Signaling Technology	Cat# 4749
anti-tubulin	Cell Signaling Technology	Cat# 38735
anti-rabbit (HRP-conjugated)	Cell Signaling Technology	Cat# 7074P2
anti-mouse (HRP-conjugated)	Cell Signaling Technology	Cat# 7076P2

Fig. S1

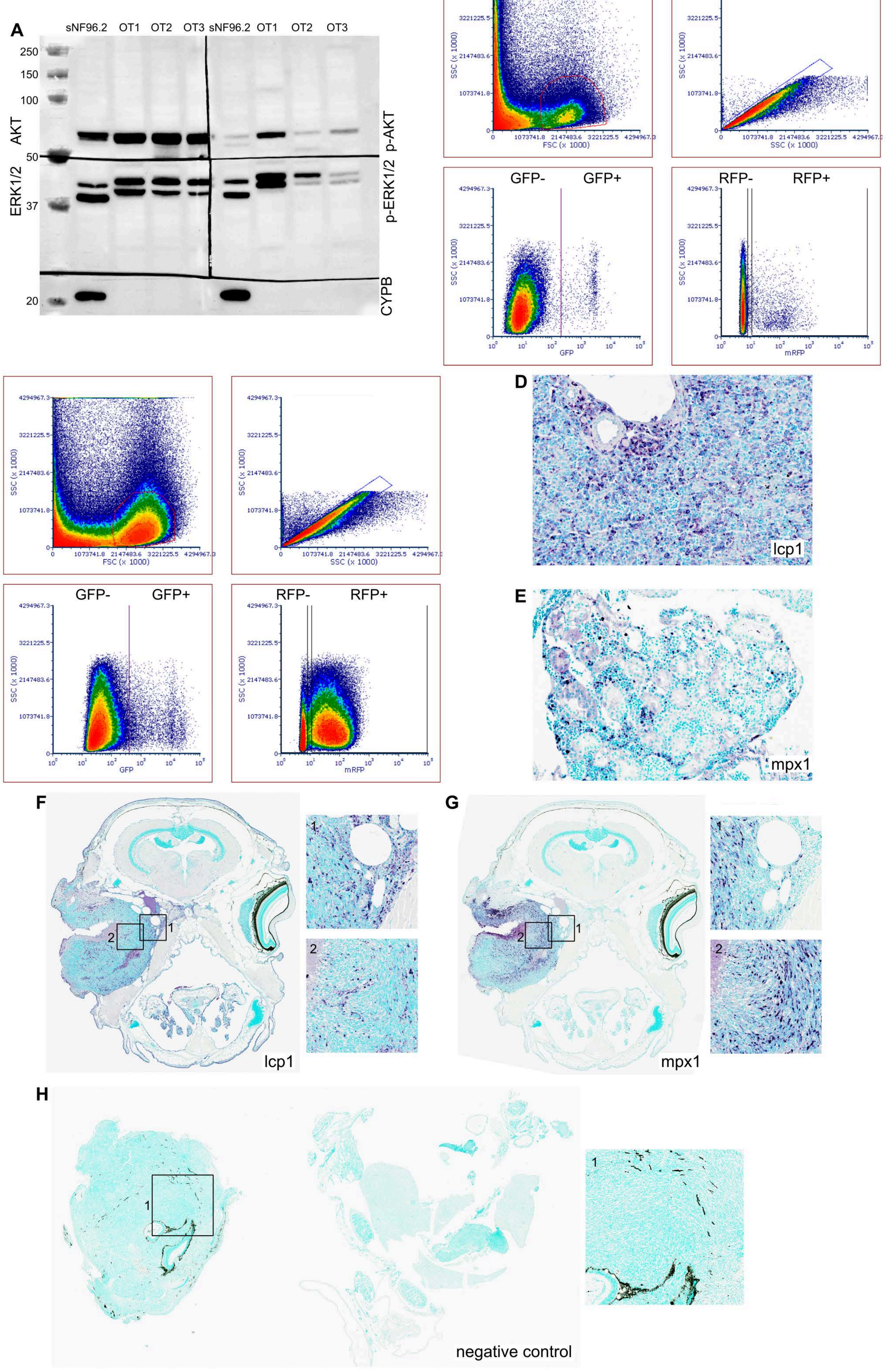

A
sNF96.2
OT1
OT2
OT3
sNF96.2
OT1
OT2
OT3
250
150
100
50
37
20
AKT
ERK1/2
p-AKT
p-ERK1/2
CYPB
B
GFP-
GFP+
RFP-
RFP+
SSC (x 1000)
FSC (x 1000)
GFP
mRFP
C
GFP-
GFP+
RFP-
RFP+
D
lcp1
E
mpx1
F
lcp1
G
mpx1
H
negative control

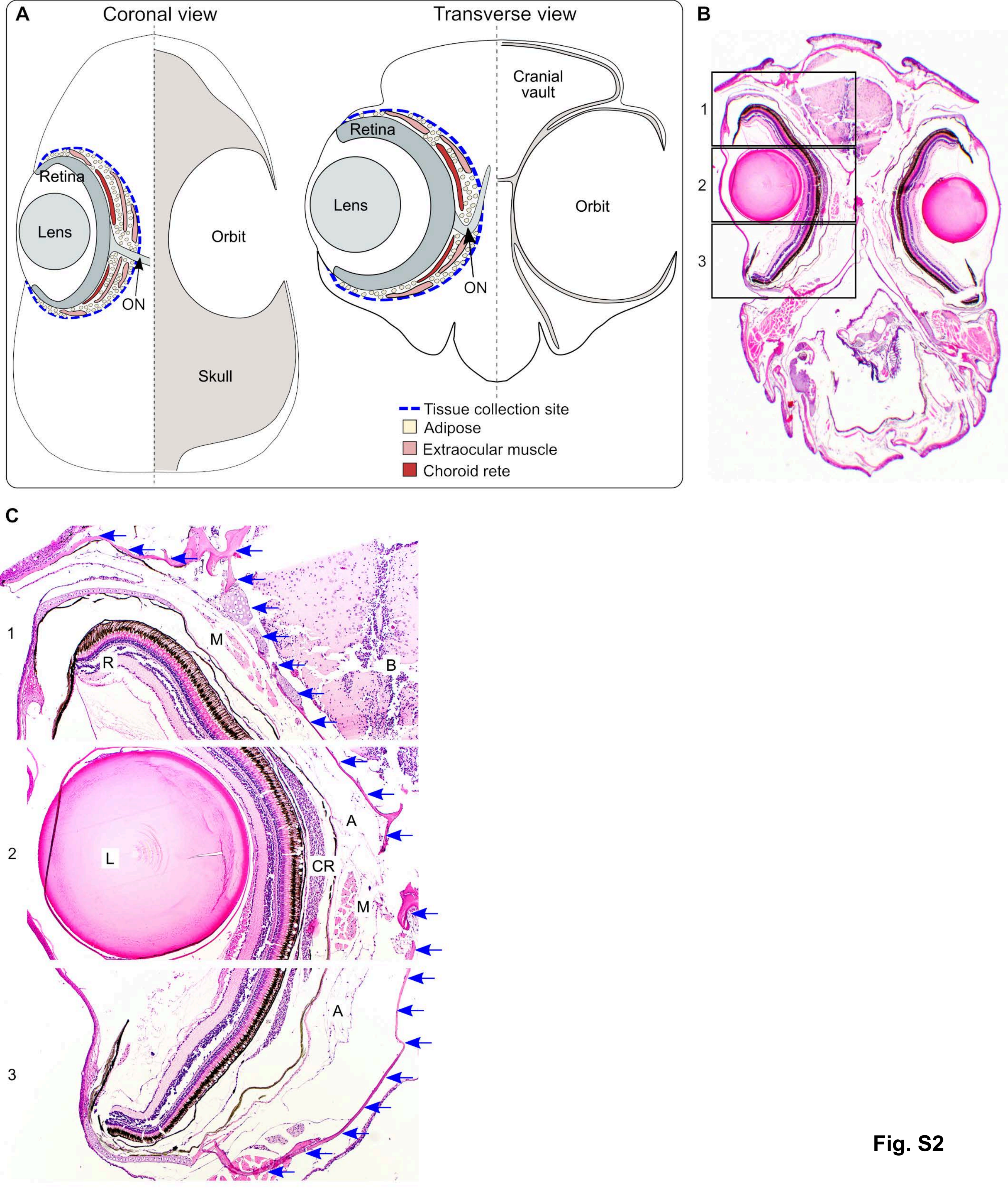

A
Coronal view
Transverse view
Retina
Lens
ON
Orbit
Skull
Cranial vault
Retina
Lens
ON
Orbit
Tissue collection site
Adipose
Extraocular muscle
Choroid rete
B
1
2
3
C
1
M
R
B
2
A
L
CR
M
3
A

Fig. S2

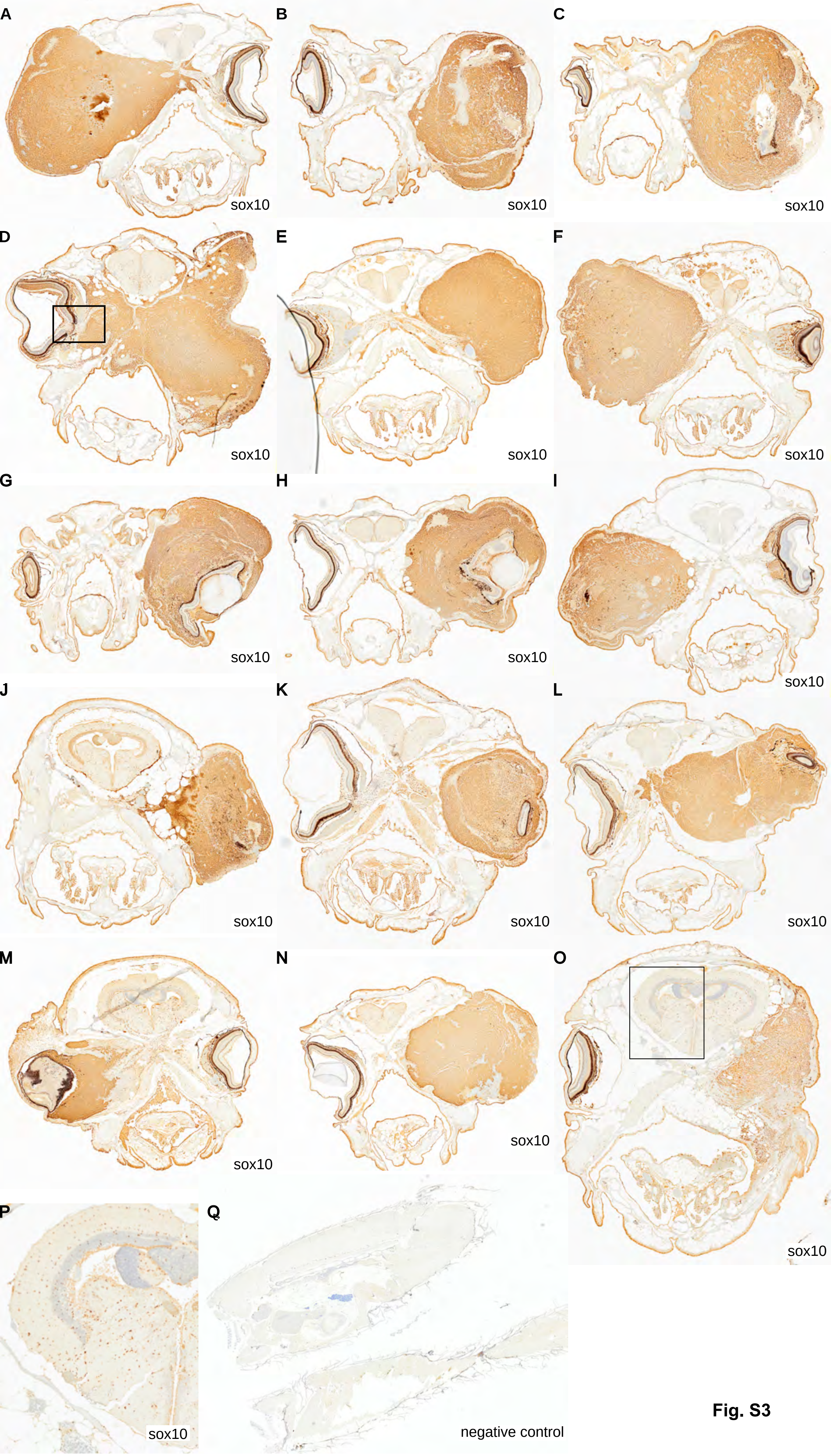
A
sox10
B
sox10
C
sox10
D
sox10
E
sox10
F
sox10
G
sox10
H
sox10
I
sox10
J
sox10
K
sox10
L
sox10
M
sox10
N
sox10
O
sox10
P
sox10
Q
negative control

Fig. S3

A Read count for indivdual samples

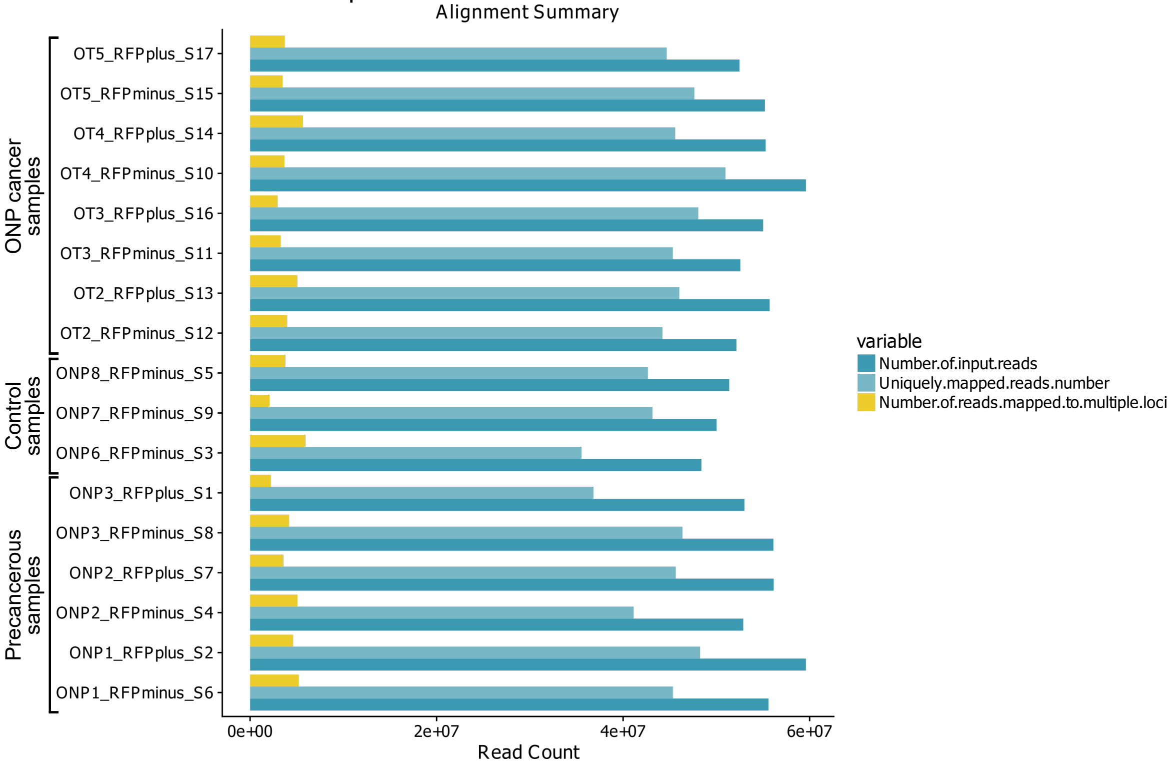

B Cluster dendrogram of experimental replicates

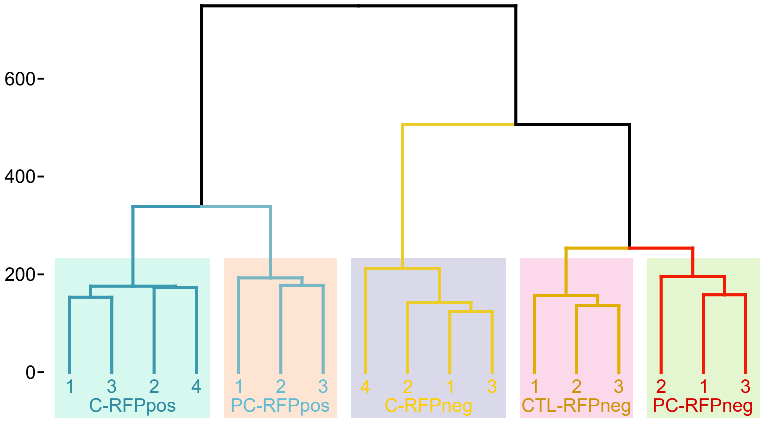

Fig. S4

A Commonly enriched canonical pathways without predicted directional activity

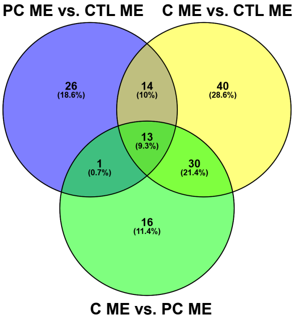

PC ME vs. CTL ME
C ME vs. CTL ME

Signaling by Rho Family GTPases
RHOGDI Signaling
Th1 and Th2 Activation Pathway
IL-13 Signaling Pathway
Chemokine Signaling
PAK Signaling
IL-12 Signaling and Production in Macrophages
T Cell Exhaustion Signaling Pathway
Antioxidant Action of Vitamin C
Virus Entry via Endocytic Pathways
Paxillin Signaling
Role of JAK1 and JAK3 in γc Cytokine Signaling
Pancreatic Adenocarcinoma Signaling
Role of Tissue Factor in Cancer

C ME vs. CTL ME
C ME vs. PC ME

Phototransduction Pathway
Phagosome Maturation
Sphingosine-1-phosphate Signaling
Circadian Rhythm Signaling
Hepatic Fibrosis / Hepatic Stellate Cell Activation
IL-15 Production
Glycolysis I
Ephrin B Signaling
G Protein Signaling Mediated by Tubby
Iron homeostasis signaling pathway
Phagosome Formation
Corticotropin Releasing Hormone Signaling
Gαq Signaling
P2Y Purigenic Receptor Signaling Pathway
GABA Receptor Signaling
Oxytocin Signaling Pathway
Gap Junction Signaling
STAT3 Pathway
CCR5 Signaling in Macrophages
Role of Macrophages, Fibroblasts and Endothelial Cells in Rheumatoid Arthritis
Systemic Lupus Erythematosus In T Cell Signaling Pathway
Apelin Muscle Signaling Pathway
Cellular Effects of Sildenafil (Viagra)
Glioblastoma Multiforme Signaling
Tight Junction Signaling
Clathrin-mediated Endocytosis Signaling
Epithelial Adherens Junction Signaling
Apelin Adipocyte Signaling Pathway
SAPK/JNK Signaling
AMPK Signaling

PC ME vs. CTL ME
C ME vs. CTL ME
C ME vs. PC ME

T Helper Cell Differentiation
RHOA Signaling
Agranulocyte Adhesion and Diapedesis
Antigen Presentation Pathway
Atherosclerosis Signaling
Molecular Mechanisms of Cancer
Granulocyte Adhesion and Diapedesis
Germ Cell-Sertoli Cell Junction Signaling
Regulation of the Epithelial-Mesenchymal Transition Pathway
Reelin Signaling in Neurons
Axonal Guidance Signaling
Semaphorin Signaling in Neurons
Human Embryonic Stem Cell Pluripotency

B Commonly enriched activated canonical pathways

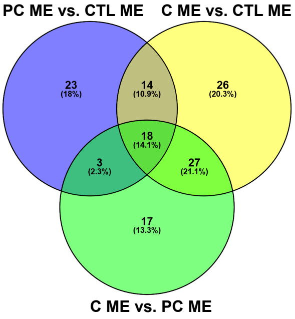

PC ME vs. CTL ME
C ME vs. CTL ME

PKCθ Signaling in T Lymphocytes
Role of NFAT in Regulation of the Immune Response
CD28 Signaling in T Helper Cells
Cell Cycle Control of Chromosomal Replication
Kinetochore Metaphase Signaling Pathway
Natural Killer Cell Signaling
Crosstalk between Dendritic Cells and Natural Killer Cells
IL-7 Signaling Pathway
Dendritic Cell Maturation
GP6 Signaling Pathway
Th1 Pathway
IL-9 Signaling
Renin-Angiotensin Signaling
Cholecystokinin/Gastrin-mediated Signaling

C ME vs. CTL ME
C ME vs. PC ME

TREM1 Signaling
Role of Hypercytokinemia/hyperchemokinemia in the Pathogenesis of Influenza
Type I Diabetes Mellitus Signaling
Pyroptosis Signaling Pathway
Colanic Acid Building Blocks Biosynthesis
Macropinocytosis Signaling
Remodeling of Epithelial Adherens Junctions
Osteoarthritis Pathway
Actin Cytoskeleton Signaling
Tumor Microenvironment Pathway
Apelin Endothelial Signaling Pathway
Induction of Apoptosis by HIV1
FcγRIIB Signaling in B Lymphocytes
HIF1α Signaling
Necroptosis Signaling Pathway
Wound Healing Signaling Pathway
N-acetylglucosamine Degradation II
Integrin Signaling
Role of PKR in Interferon Induction and Antiviral Response
FAT10 Signaling Pathway
Endothelin-1 Signaling
RAC Signaling
IL-8 Signaling
TWEAK Signaling
Role of MAPK Signaling in Promoting the Pathogenesis of Influenza
Gαi Signaling
CCR3 Signaling in Eosinophils

PC ME vs. CTL ME
C ME vs. CTL ME
C ME vs. PC ME

Systemic Lupus Erythematosus In B Cell Signaling Pathway
TEC Kinase Signaling
NF-κB Signaling
Phospholipase C Signaling
Production of Nitric Oxide and Reactive Oxygen Species in Macrophages
Interferon Signaling
Actin Nucleation by ARP-WASP Complex
Neuroinflammation Signaling Pathway
Fcγ Receptor-mediated Phagocytosis in Macrophages and Monocytes
Glioma Invasiveness Signaling
Thrombin Signaling
fMLP Signaling in Neutrophils
CXCR4 Signaling
ILK Signaling
Leukocyte Extravasation Signaling
Regulation of Actin-based Motility by Rho
Ephrin Receptor Signaling
Activation of IRF by Cytosolic Pattern Recognition Receptors

C Commonly enriched inhibited canonical pathways

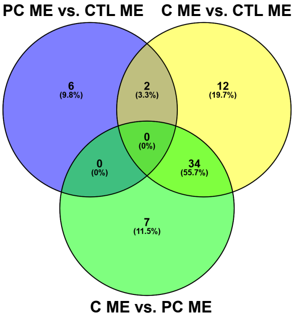

PC ME vs. CTL ME
C ME vs. CTL ME

PD-1, PD-L1 cancer immunotherapy pathway
Regulation Of The Epithelial Mesenchymal Transition In Development Pathway

C ME vs. CTL ME
C ME vs. PC ME

Calcium Signaling
White Adipose Tissue Browning Pathway
CREB Signaling in Neurons
Synaptogenesis Signaling Pathway
Netrin Signaling
Gustation Pathway
SNARE Signaling Pathway
Factors Promoting Cardiogenesis in Vertebrates
Dopamine-DARPP32 Feedback in cAMP Signaling
GPCR-Mediated Nutrient Sensing in Enteroendocrine Cells
Neuropathic Pain Signaling In Dorsal Horn Neurons
WNT/Ca+ pathway
Oxytocin In Spinal Neurons Signaling Pathway
Neurovascular Coupling Signaling Pathway
Synaptic Long Term Depression
Cardiac β-adrenergic Signaling
Glutamate Receptor Signaling
Endocannabinoid Neuronal Synapse Pathway
Huntington's Disease Signaling
Dilated Cardiomyopathy Signaling Pathway
Role of NFAT in Cardiac Hypertrophy
GNRH Signaling
Sperm Motility
Protein Kinase A Signaling
Gαs Signaling
Oxytocin In Brain Signaling Pathway
G-Protein Coupled Receptor Signaling
Opioid Signaling Pathway
Breast Cancer Regulation by Stathmin1
FAK Signaling
Cardiac Hypertrophy Signaling (Enhanced)
Synaptic Long Term Potentiation
Semaphorin Neuronal Repulsive Signaling Pathway
LXR/RXR Activation

Fig. S5

A Candidate gene testing in human MPNST samples

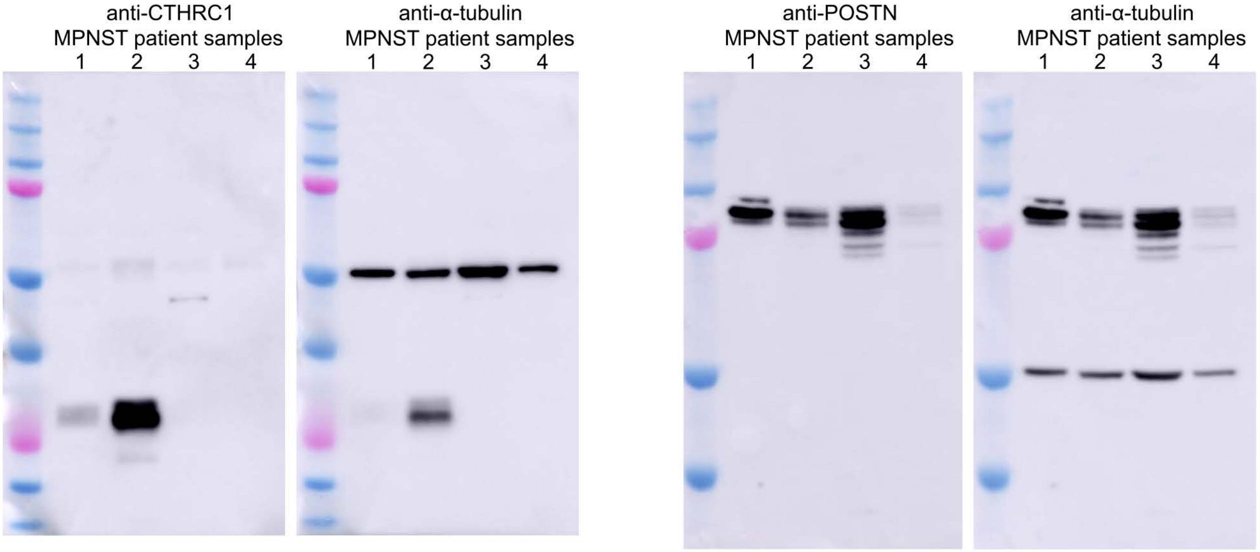

B Immunohistochemistry for POSTN
Human peripheral nerve tumor tissue microarray

A1-E4: Malignant
E5-I4: Benign
I5-J10: Normal nerve (not scored)
‡ TMA standard internal control (not scored)

Scoring for POSTN distribution

% positive area	Score
<10% sample	0
10-25%	1
25-50%	2
50-75%	3
75-100%	4

Scoring for POSTN intensity

Labeling intensity	Score
Minimal/absent	0
Weak	1
Moderate	2
Intense	3

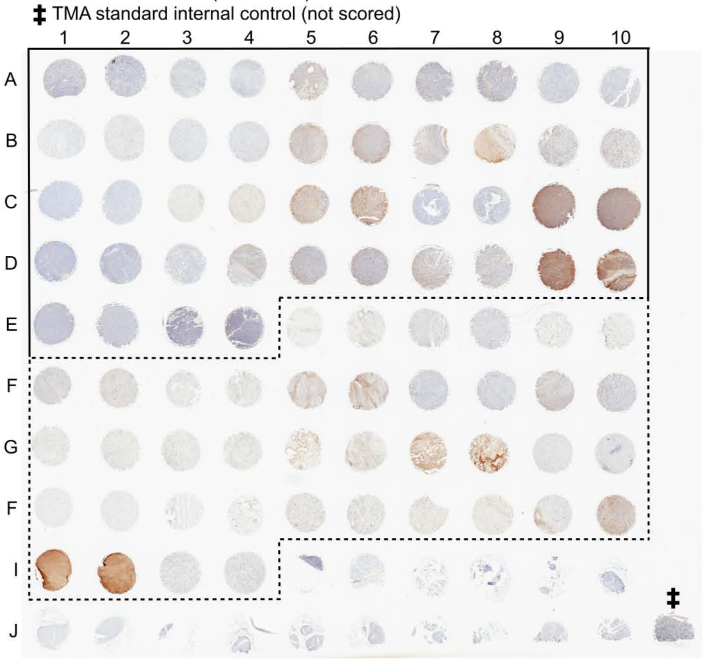

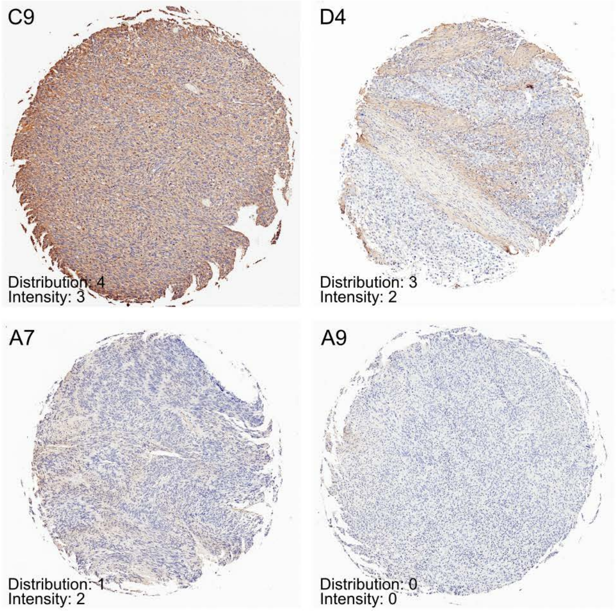

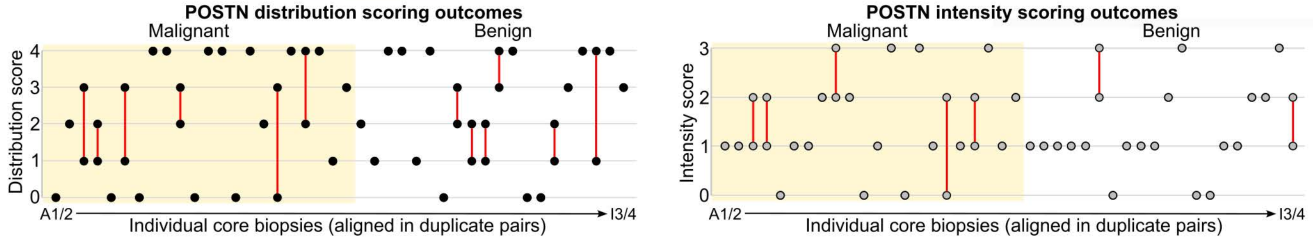

C Immunohistochemistry for POSTN
Control specimens

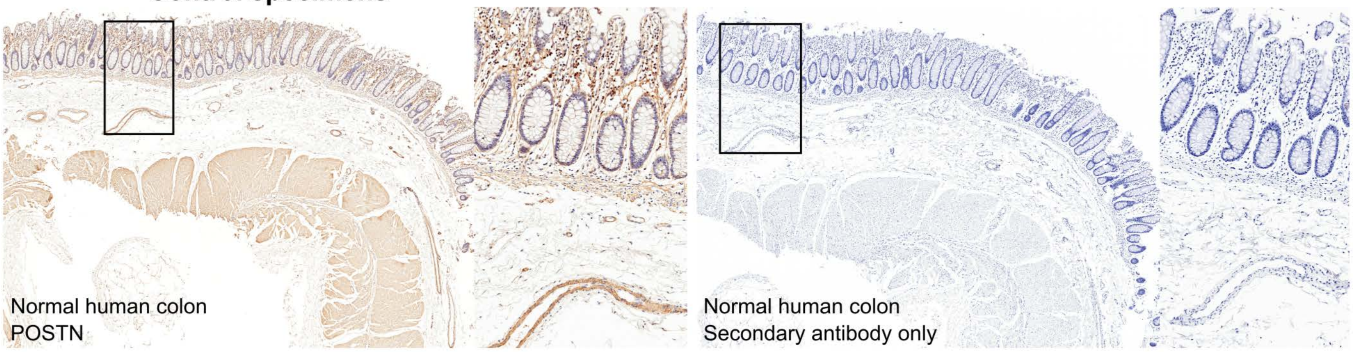

Fig. S6

A Probe validation for *postna* and *postnb*

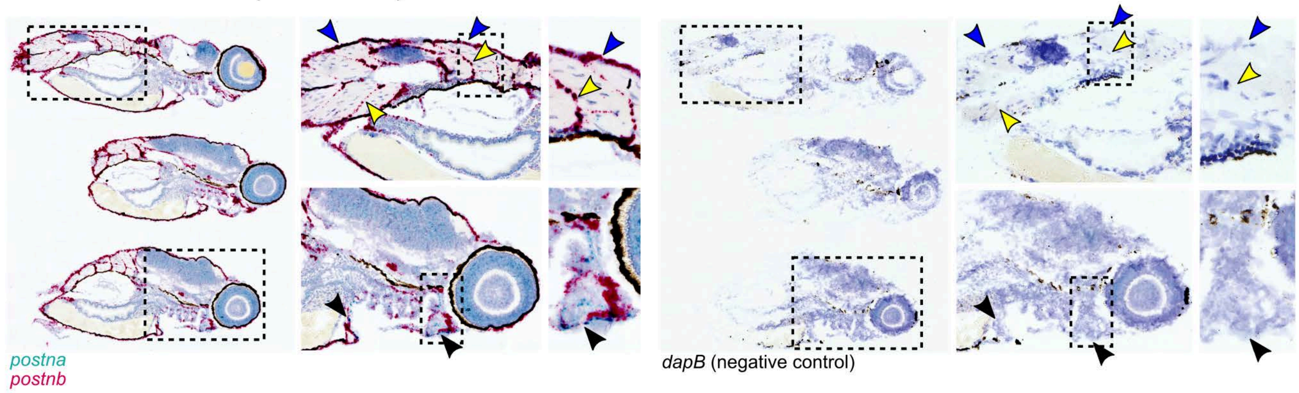

B Moderate *postna* and *postnb* expression in a zebrafish ONP cancer specimen

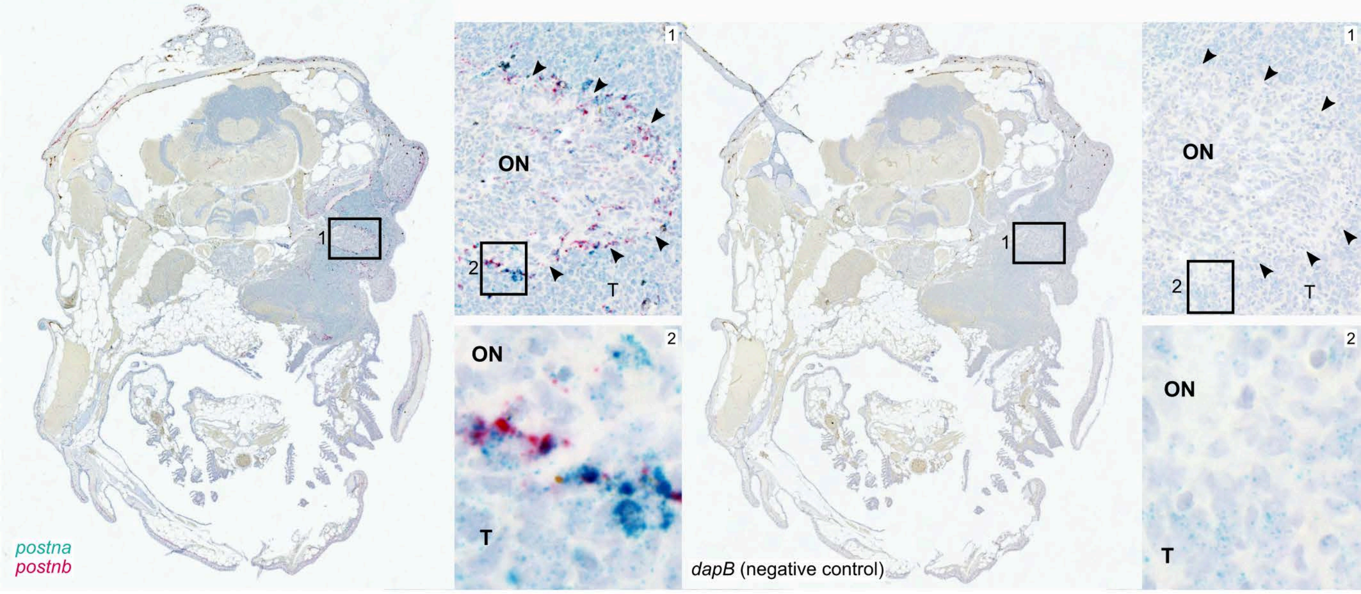

C Rare *postna* expression and low *postnb* expression in a zebrafish ONP cancer specimen

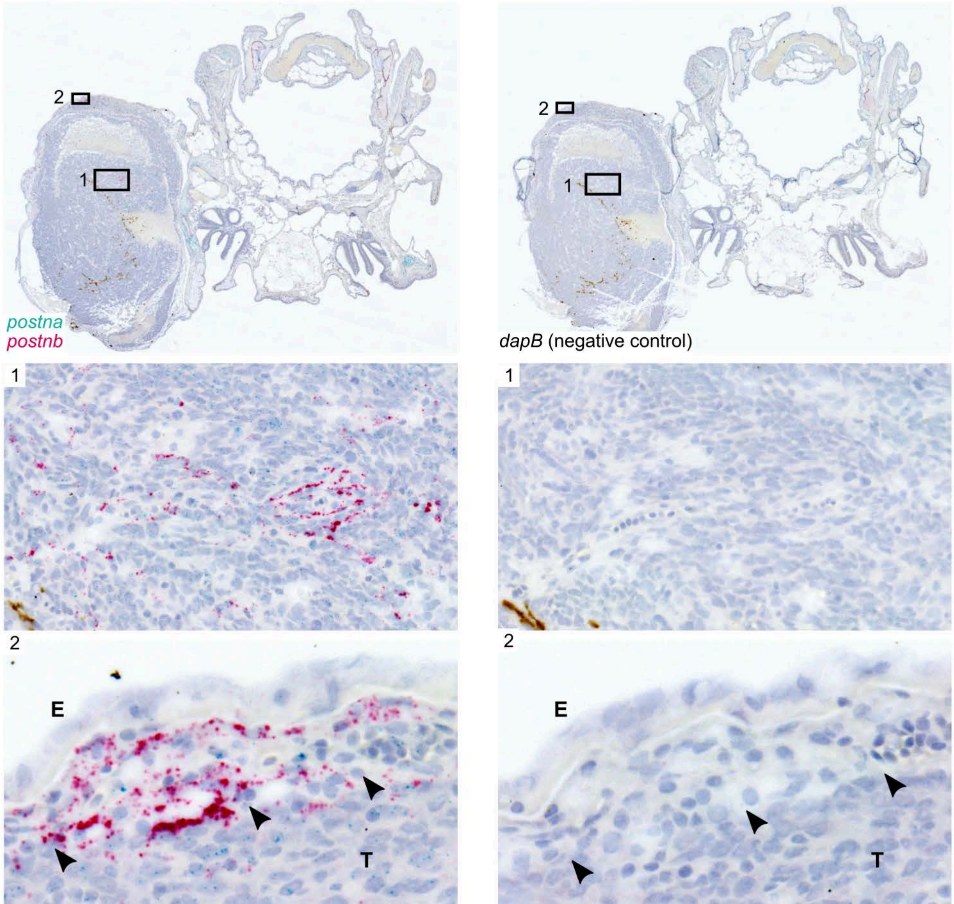

Fig. S7

Fig. S8

A Timeline for POSTN knockdown experimental assays in MPNST cells

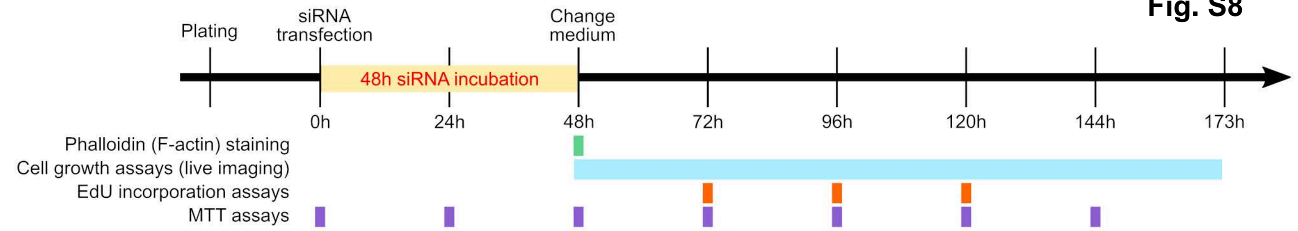

B POSTN knockdown persists up to 144 hours post-transfection

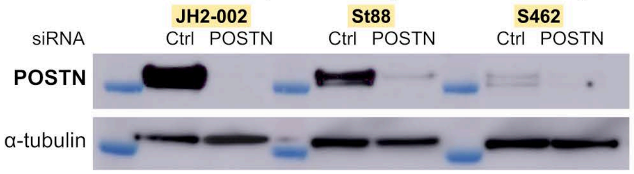

C POSTN knockdown reduces MPNST cell size and alters cytoskeletal morphology

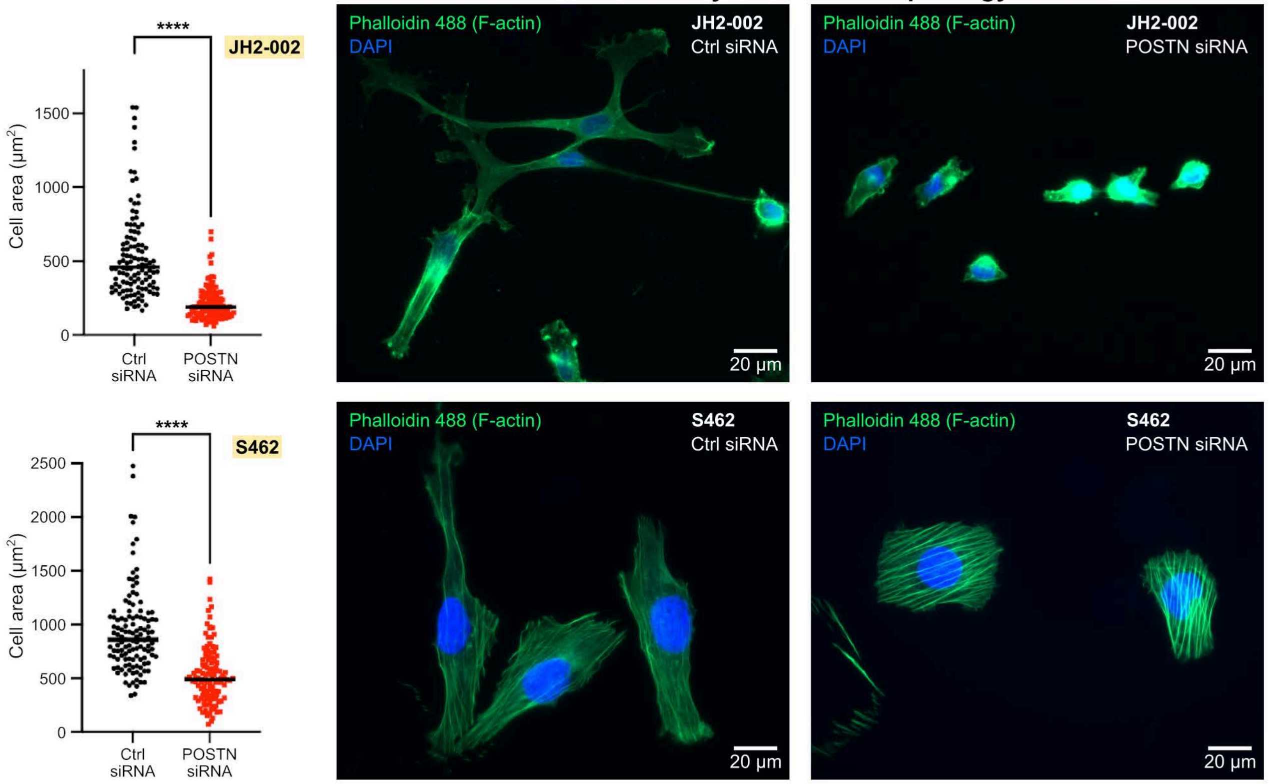

D POSTN knockdown impairs MPNST growth by inducing cytotoxicity and reducing proliferation

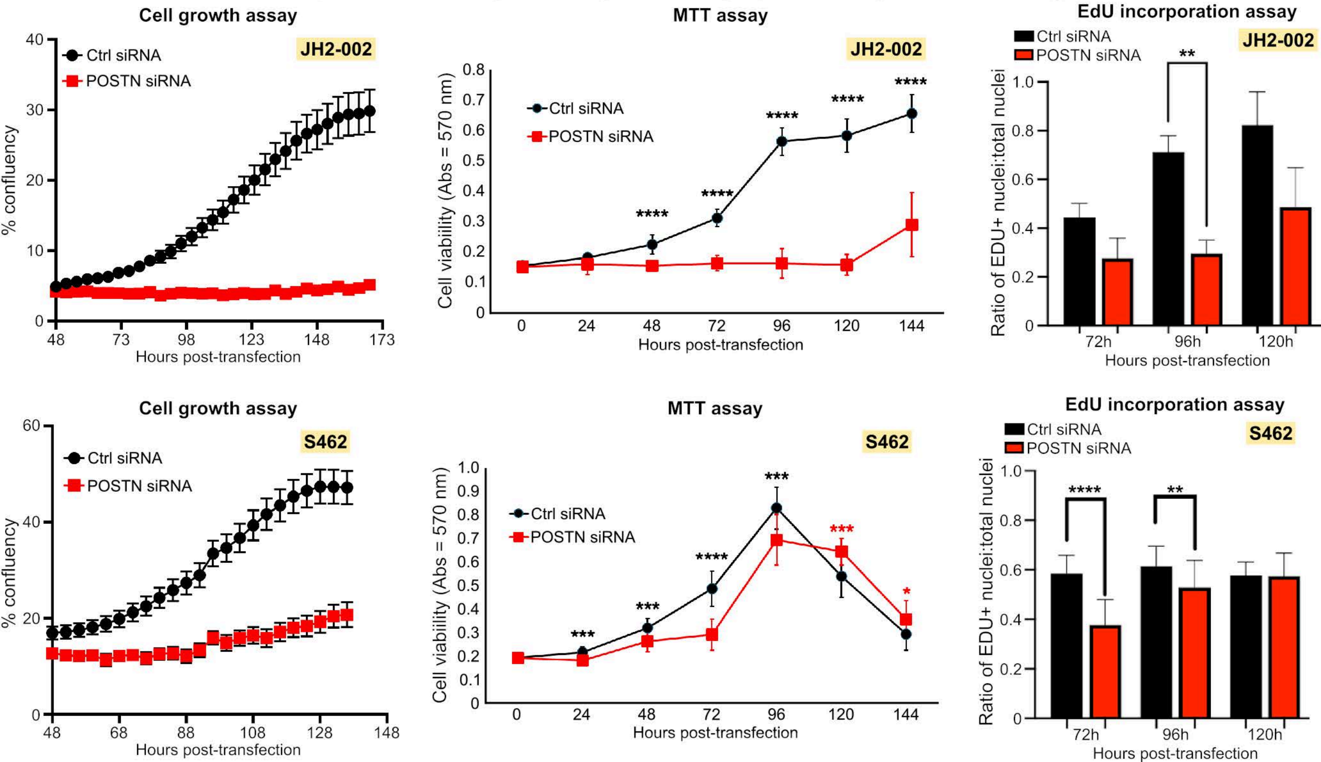

Fig. S9

A POSTN expression in human MPNST cells

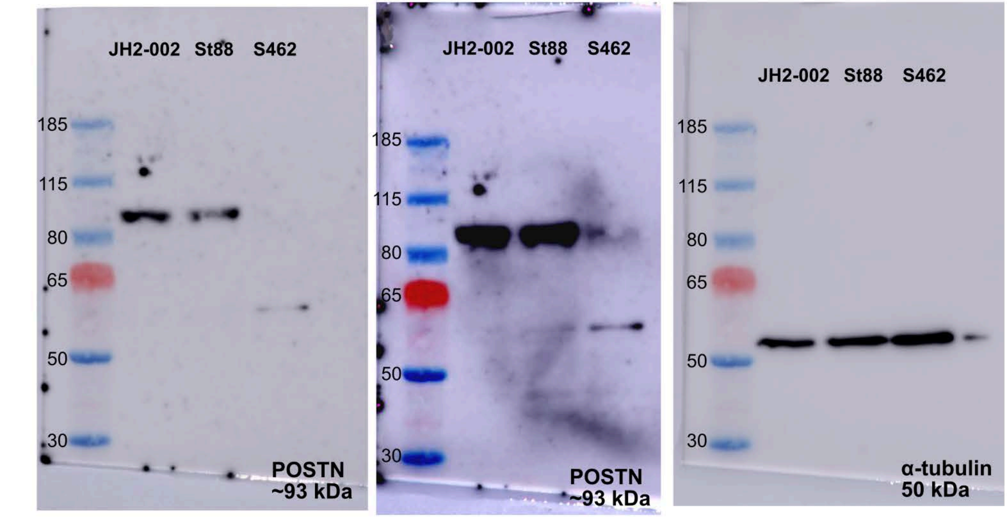

B POSTN knockdown persists up to 144 hours post-transfection

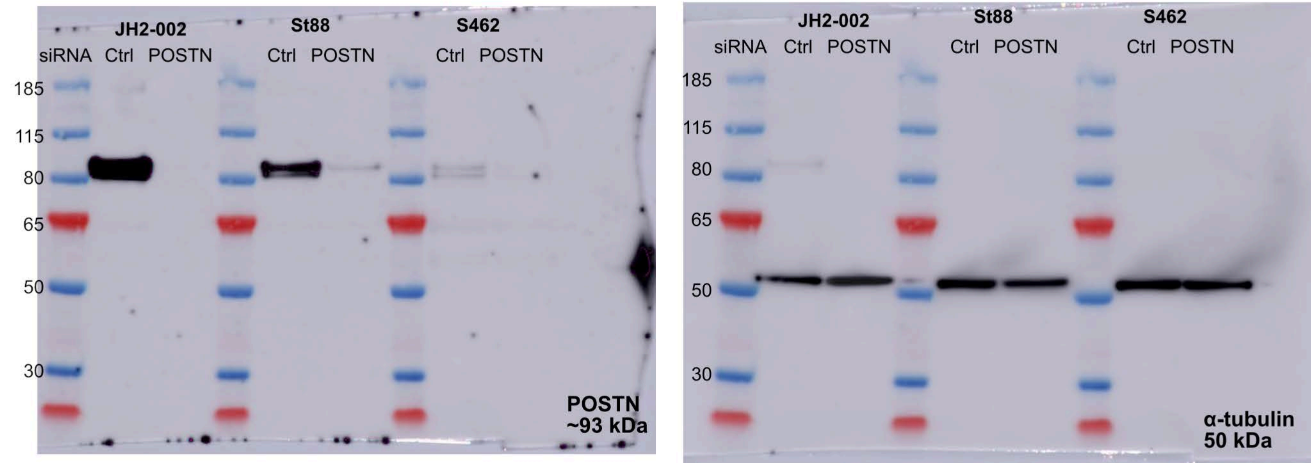

A POSTN knockdown and integrin receptor expression in human MPNST cells

Fig. S10

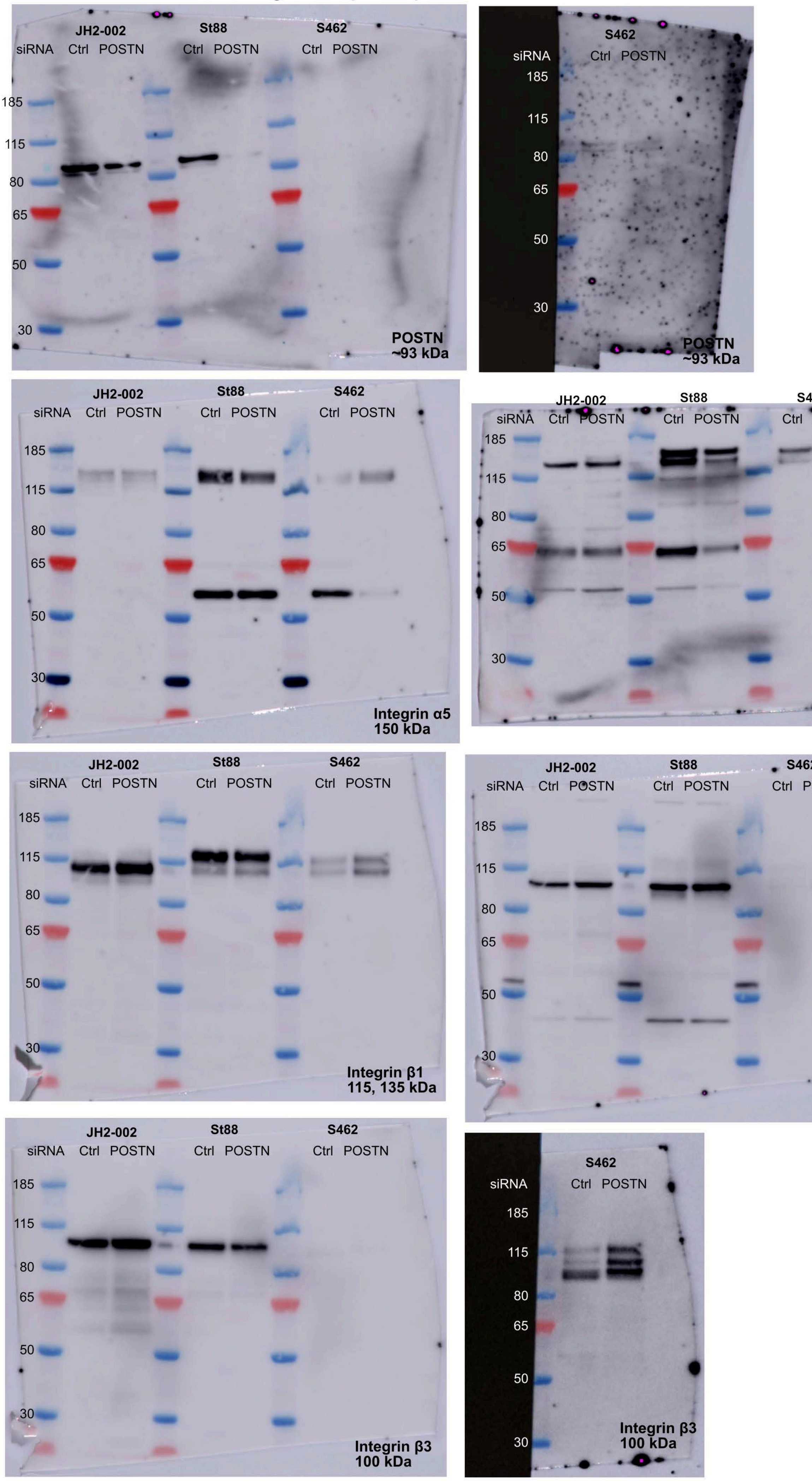

B Expression of loading control protein α-tubulin in human MPNST cells for blots shown in panel B

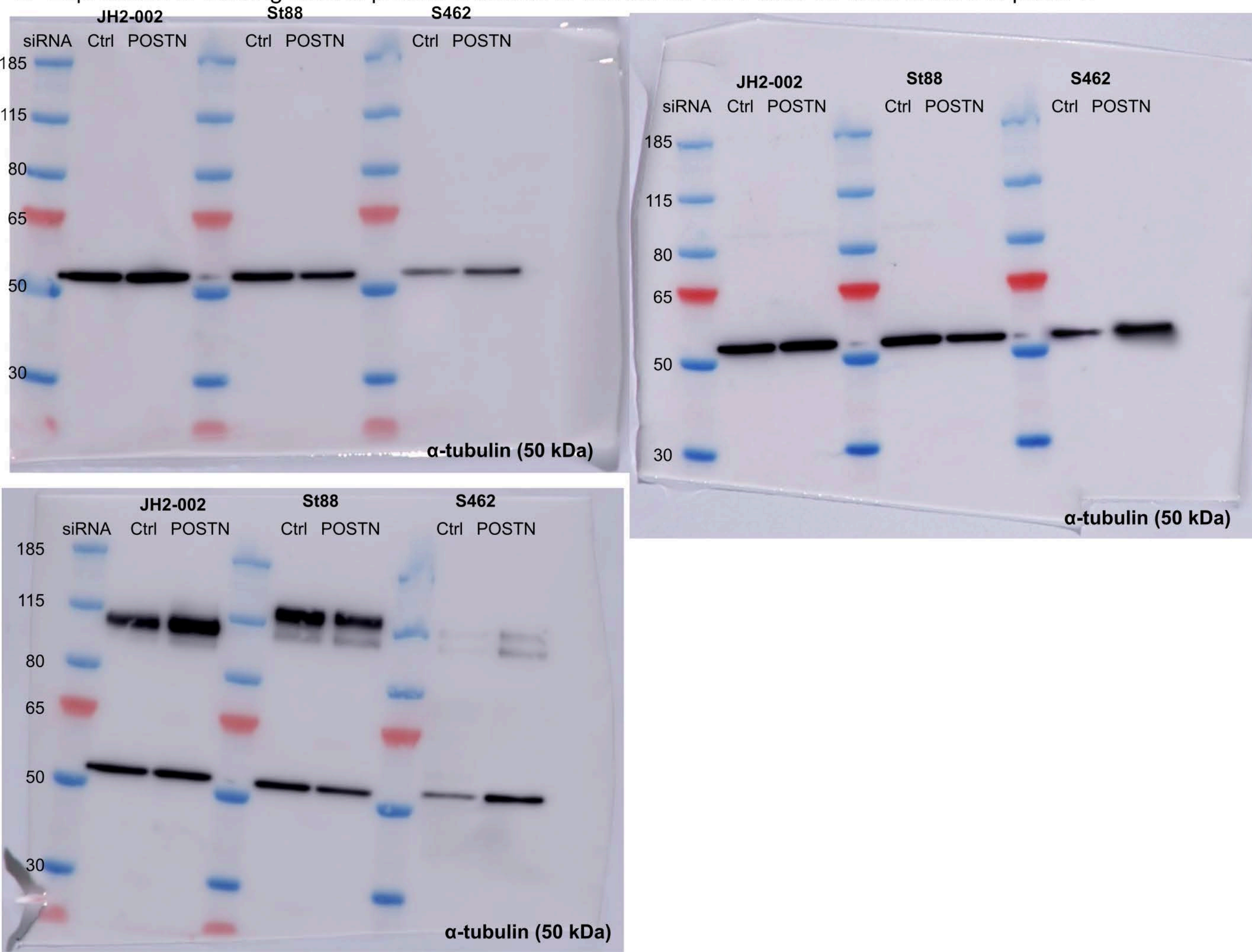

Supplement: Supplementary file 1 — Combined supplementary files [file 41388_2024_3210_MOESM1_ESM.pdf]
